# PDK1 promotes ovarian cancer metastasis by modulating tumor-mesothelial adhesion, invasion, and angiogenesis via α5β1 integrin and JNK/IL-8 signaling

**DOI:** 10.1038/s41389-020-0209-0

**Published:** 2020-02-18

**Authors:** Michelle K. Y. Siu, Yu-xin Jiang, Jing-jing Wang, Thomas H. Y. Leung, Siew Fei Ngu, Annie N. Y. Cheung, Hextan Y. S. Ngan, Karen K. L. Chan

**Affiliations:** 1 Departments of Obstetrics and Gynaecology, University of Hong Kong, Special Administrative Region of China, Hong Kong, Hong Kong; 2 Department of Pathology, University of Hong Kong, Special Administrative Region of China, Hong Kong, Hong Kong

**Keywords:** Metastasis, Cell adhesion, Cell migration, Cell signalling, Ovarian cancer

## Abstract

Ovarian cancer is the most lethal gynecological malignancies owing to the lack of definitive symptoms until development of widespread metastases. Identification of novel prognostic and therapeutic targets is therefore an urgent need to improve survival. Here, we demonstrated high expression of the mitochondrial gatekeeping enzyme, pyruvate dehydrogenase kinase 1 (PDK1), in both clinical samples and cell lines of ovarian cancer. PDK1 expression was significantly associated with metastasis, reduced chemosensitivity, and poor overall and disease-free survival, and further highlighted as an independent prognostic factor. Silencing of PDK1 retarded lactate production, ovarian cancer cell adhesion, migration, invasion, and angiogenesis, and consequently metastasis, concomitant with decreased α5β1 integrin expression. Phospho-kinase array profiling and RNA sequencing analyses further revealed reduction of JNK activation and IL-8 expression in PDK1-depleted cells. Conversely, PDK1 overexpression promoted cell adhesion via modulation of α5β1 integrins, along with cell migration, invasion, and angiogenesis through activation of JNK/IL-8 signaling. PDK1 depletion additionally hindered tumor growth and dissemination in nude mice in vivo. Importantly, PDK1 levels were upregulated upon treatment with conditioned medium from omental tissues, which in turn promoted metastasis. Our findings suggest that PDK1, which is regulated by the tumor microenvironment, controls lactate production and promotes ovarian cancer cell metastasis via modulation of α5β1 integrin and JNK/IL-8 signaling. To our knowledge, this is the first report to demonstrate an association between PDK1 and survival in patients with ovarian cancer, supporting its efficacy as a valuable prognostic marker and therapeutic molecular target for the disease.

## Introduction

Ovarian cancer has the highest mortality rate among all gynecological malignancies worldwide^1^. Symptoms are often vague, and patients tend to present late, with extensive metastases. Despite recent advances in treatment options, the overall prognosis remains poor^2,3^. Continued efforts to identify and develop new target therapies are therefore essential. As an intra-abdominal tumor, exfoliated ovarian cancer cells detached from the primary tumor are carried by peritoneal fluid and preferentially disseminate within the peritoneal cavity^2,3^. Based on Paget’s “seed and soil” theory, the mesothelium that covers all organs within the peritoneal cavity, including omentum and peritoneum, serves as the “soil” for the “seed” ovarian cancer cells to attach and invade. These steps, together with induction of angiogenesis, contribute to the formation of metastatic foci^2,3^.

Altered glucose metabolism is considered a hallmark of cancer^4–6^. One of the major characteristics of the Warburg effect (aerobic glycolysis) is that pyruvate is converted to lactate in the cytoplasm instead of being further oxidized in the mitochondria by pyruvate dehydrogenase (PDH), the mitochondrial gatekeeper^7–9^. Thus, blockage of PDH activity is critical in achieving the Warburg effect. PDH has been identified as an E1 enzyme, which together with E2 and E3 enzymes, forms the pyruvate dehydrogenase complex (PDC). PDH activity is regulated by pyruvate dehydrogenase kinase (PDK) and pyruvate dehydrogenase phosphatase (PDP). PDKs are Ser/Thr kinases that phosphorylate the α-subunit of PDH, leading to inactivation of PDH, and consequently, PDC. Conversely, dephosphorylation of PDH by PDP restores PDC activity. PDKs are thus defined as gatekeeping enzymes that regulate the shunt of pyruvate into the mitochondria^10,11^.

Four PDK isoenzymes (PDK1–4) have been identified in humans, with PDK1 being the best-studied isoenzyme^10–12^. The metabolic switch mediated by PDK1 has been shown to support malignant phenotypes in vitro such as head-and-neck squamous cell carcinoma (HNSC) resistance to hypoxia-induced cell death^13^, breast cancer cell anoikis resistance^14^, oncogene-induced senescence in melanomas^15^, and breast cancer stem cell reprogramming^16^. Knockdown of PDK1 is reported to impede tumor growth in nude mice in HNSC, melanoma, and breast cancer cells^13,15,16^. Tyrosine phosphorylation activates PDK1 to promote the Warburg effect and in vivo tumor growth in leukemia and lung cancer cells^17^. Moreover, high PDK1 expression is correlated with poor prognosis in HNSC^18^ and gastric cancer^19^.

At present, little is known about altered glucose metabolism patterns in ovarian cancer. Increased lactate levels in both primary and metastatic ovarian cancer relative to their normal ovarian tissue counterparts has been documented^20^. PDK1 was overexpressed in the highly glycolytic human ovarian cancer cell line OC316 compared with the less glycolytic cell line IGROV-1^21^. Dicumarol, a coumarin compound, has been found to inhibit PDK1 and suppress ovarian cancer tumor growth in vivo^22^. A recent study demonstrated PDK1 contributes to cisplatin resistance of ovarian cancer through EGFR activation and promotes epithelial–mesenchymal transition^23^. In this study, we focused on the clinical significance, functional roles, and downstream mechanisms of PDK1 in ovarian cancer. The effects of conditioned medium derived from ovarian cancer-associated fibroblasts (CAF-CM) and omentum (OCM) on PDK1 expression were also assessed.

## Results

### Increased expression of PDK1 is associated with ovarian cancer metastasis and poor patient prognosis

**Fig. 1:**
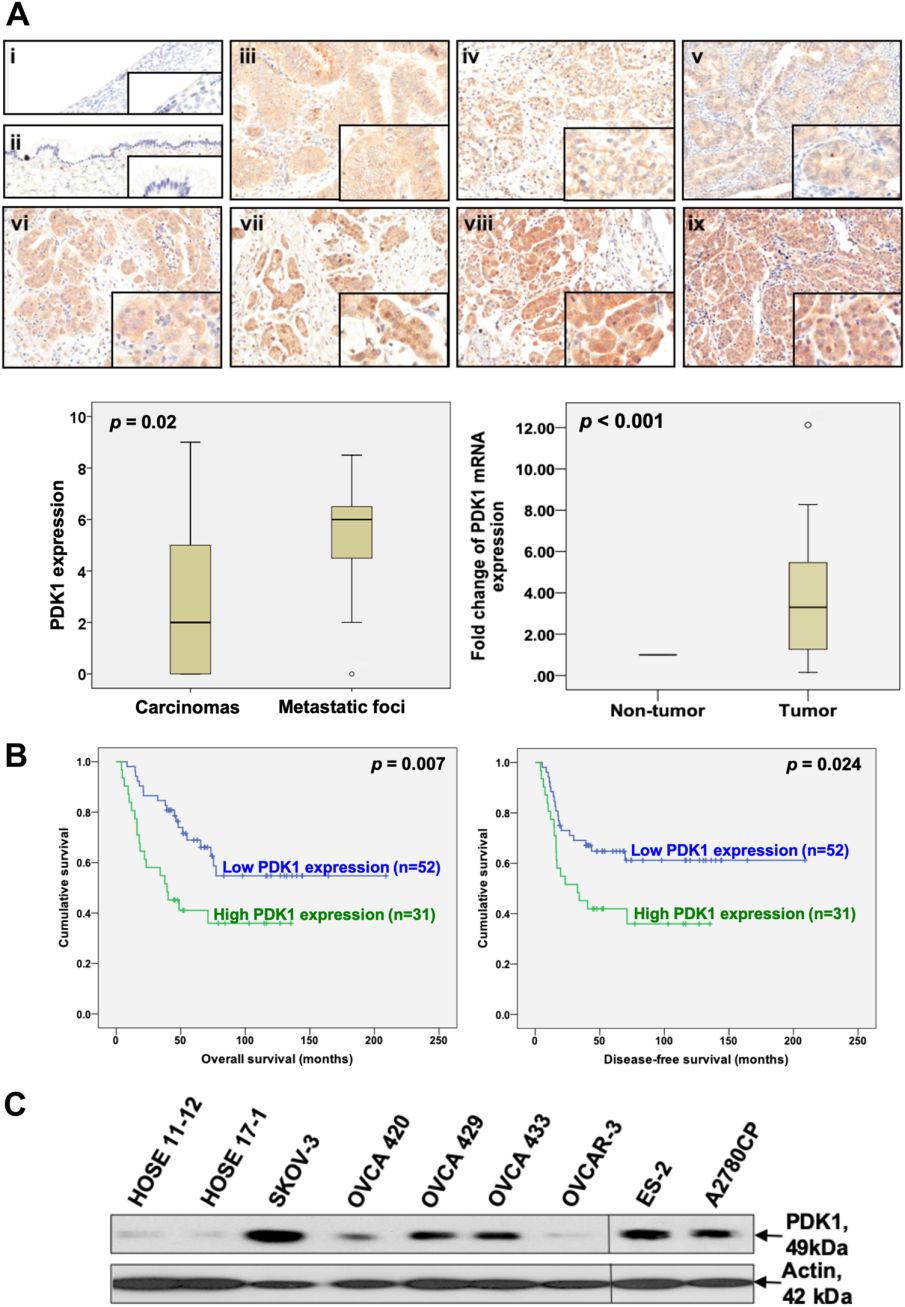
Overexpressed PDK1 in ovarian cancer is associated with tumor metastasis and poor survival. **a** Upper: Immunohistochemical staining of PDK1 in serous (i) and mucinous (ii) benign cystadenomas, mucinous (iii), clear cell (iv), endometrial (v) carcinomas, primary serous carcinomas (vi), and matched metastatic foci (vii, viii, and ix). The insets highlight regions with higher magnification. Lower left: Immunoscoring of PDK1 in primary carcinomas and corresponding metastatic foci. The staining score for replicate metastatic lesions was averaged and considered as one value for that case for statistical purposes. Lower right: qPCR analysis of PDK1 mRNA in ovarian cancer samples (tumor) and their corresponding normal counterparts (non-tumor). **b** Kaplan–Meier overall (left panel) and disease-free (right panel) survival curves with respect to PDK1 expression (cutoff at mean). **c** Protein expression of PDK1 in HOSE (HOSE 11-12 and HOSE 17-1) and ovarian cancer cell lines (SKOV-3, OVCA 420, OVCA 429, OVCA 433, OVCAR-3, ES-2, and A2780CP) determined via immunoblot analysis.

PDK1 protein expression in 130 paraffin-embedded tissue samples was evaluated via immunohistochemistry. PDK1 was primarily localized in the cytoplasm (Fig. 1a). PDK1 staining was moderate-to-strong in ovarian cancers, in contrast to barely detectable staining in benign cystadenomas (Fig. 1a, upper). Immunoreactivity to PDK1 was significantly higher in ovarian cancers than benign cystadenomas (*P* < 0.001, Supplementary Table 1). Moreover, higher PDK1 immunoreactivity was observed in metastatic foci than the corresponding primary carcinomas (*P* = 0.02, Fig. 1a, lower left). High PDK1 immunoreactivity was markedly associated with resistance to chemotherapy (*P* = 0.028, Supplementary Table 1) and shorter overall (*P* < 0.05) and disease-free (*P* < 0.05) survival (Fig. 1b). In contrast, we observed no significant association between disease stage, tumor grade, or histological type of cancer and PDK1 expression. In multivariate analysis, PDK1 expression, disease stage, grade, and chemosensitivity were significant independent predictors of overall survival (all *P* < 0.05, Supplementary Table 2). qPCR analyses additionally revealed significantly higher PDK1 mRNA levels in ovarian cancers relative to their corresponding non-tumor counterparts from 20 paired clinical samples (*P* < 0.05, Fig. 1a, lower right). PDK1 expression was consistently upregulated in ovarian cancer, compared with nonmalignant human ovarian epithelial cell lines (HOSE) (Fig. 1c).

### PDK1 regulates lactate production

**Fig. 2:**
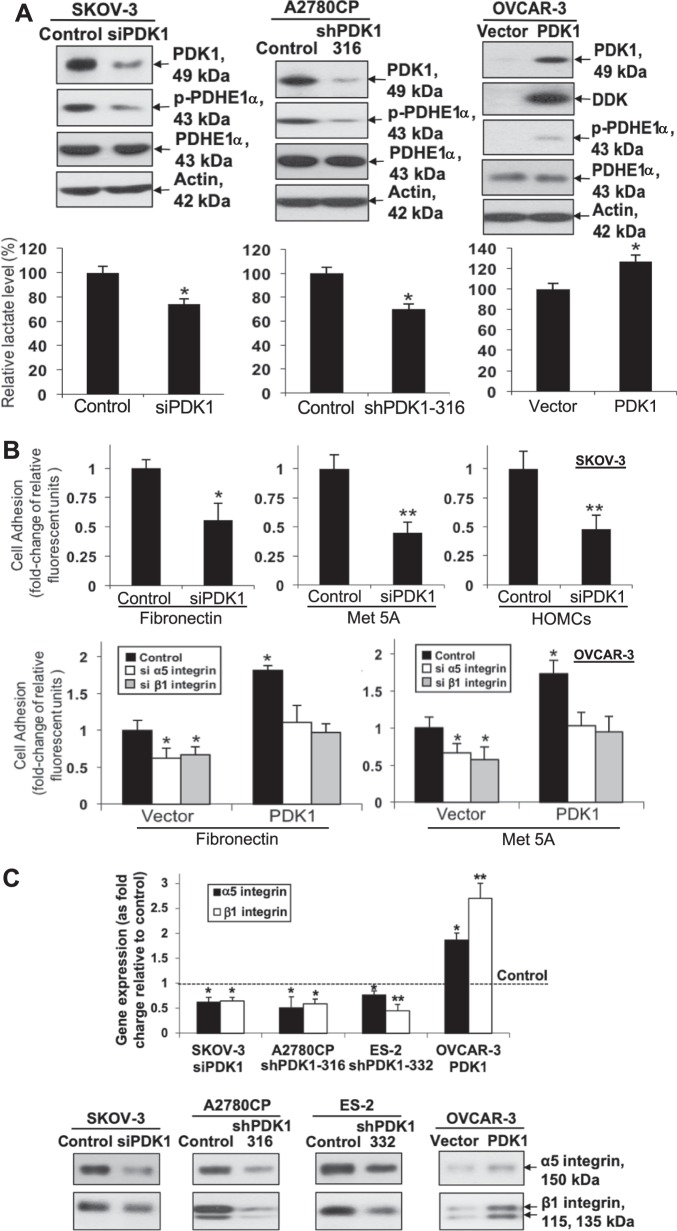
Knockdown of PDK1 impedes while overexpression of PDK1 promotes lactate production. PDK1 regulates cell adhesion via α5 and β1 integrin expression. **a** Upper left: Transient knockdown of PDK1 (via siPDK1) protein expression in SKOV-3, assessed using immunoblot. Upper middle: Stable knockdown of PDK1 protein expression in A2780CP (shPDK1-316), assessed using western blot. Left and middle: p-PDHE1α and PDHE1α protein, and fold change of lactate levels in PDK1-silenced and control SKOV-3 and A2780CP cells determined via immunoblot analysis and lactate colorimetric assay, respectively; Bars: mean ± SD of three experiments; *P < 0.05; Mann–Whitney test. Media from cultured cells were collected and used for measuring lactate levels. Upper right: Immunoblot analysis of exogenous DDK-tagged PDK1 and p-PDHE1α expression in OVCAR-3 cells stably transfected with control vector pCMV6-DDK or DDK-tagged PDK1. Lower right: fold change in lactate levels, compared with control OVCAR-3 cells expressing PDK1; Bars: mean ± SD of three experiments; *P < 0.05; Mann–Whitney test. Media from cultured cells were collected and used for measuring lactate levels. **b** Upper: Adhesion assay with control SKOV-3 and PDK1-depleted cells. Ovarian cancer cells labeled with 5 μM calcein-AM were added on top of 96-well plates coated with fibronectin, mesothelial MeT-5A cells, or primary human adult omentum-derived mesothelial cells. After 45 min, total fluorescence and fluorescence after washing off were measured. The percentage of bound cells was calculated based on fluorescence after washing, compared with total fluorescence. Cell adhesion to fibronectin, mesothelial Met-5A cells, or primary human adult omentum-derived mesothelial cells presented as fold change, compared with control; Bars: mean ± SD of three experiments; *P < 0.05; **P < 0.005, Mann–Whitney test. Lower: Adhesion assay with control and PDK1-overexpressing OVCAR-3 cells transfected with siRNAs specific for α5 or β1 integrin. Cell adhesion presented as fold change, compared with control; Bars: mean ± SD of three experiments; **P < 0.005; Mann–Whitney test. **c** qPCR (upper) and immunoblot (lower) analysis of α5 and β1 integrin protein expression in control, siPDK1 SKOV-3, shPDK1-316 A2780CP, shPDK1-332 ES-2, and OVCAR-3 cells expressing PDK1.

Next, we examined the cell metabolic effects of PDK1 on ovarian cancer. For transient knockdown, PDK1 siRNA induced a significant reduction in both mRNA and protein levels in SKOV-3 and A2780CP cells relative to control cells (Fig. 2a, upper left; Supplementary Fig. 1A). For stable knockdown, we detected stable knockdown of PDK1 in A2780CP (shPDK1-316 and shPDK1 207) and ES-2 cells (shPDK1-332 and shPDK1 226) (Fig. 2a, upper middle; Supplementary Fig. 1B). Lactate levels in media from cultured cells were measured. Both transient (SKOV-3) and stable (A2780CP) PDK1-silenced cells displayed significantly reduced lactate levels, compared with control cells, together with reduced phosphorylation of pyruvate dehydrogenase E1-alpha subunit (PDHE1α) (Fig. 2a, lower left and middle; Supplementary Fig. 1C). Conversely, in OVCAR-3 cells stably transfected with DDK-tagged PDK1 plasmid or control vector, ectopic overexpression of PDK1 was detected via western blot using anti-PDK1 and anti-DDK antibodies (Fig. 2a, upper right). PDK1-overexpressing cells displayed increased p-PDHE1α and lactate levels (Fig. 2a, right).

### PDK1 augments cell adhesion via regulation of α5 and β1 integrin expression

In view of the significantly higher PDK1 immunoreactivity in metastatic foci compared with their corresponding primary carcinomas, we evaluated the effects of PDK1 on ovarian cancer cell adhesion to fibronectin and mesothelial cells via the cell-adhesion assay. Notably, transient knockdown of PDK1 in SKOV-3 (Fig. 2b, upper) and ectopic expression of PDK1 in OVCAR-3 (Fig. 2b, lower) significantly impeded and enhanced cell adherence to fibronectin and mesothelial cells, respectively. Next, the downstream targets mediating the effects of PDK1 on adhesion were examined. When disseminated primary ovarian cancer cells target the omentum or peritoneum, they initially interact with mesothelial cells through integrin receptors^3,24,25^. Remarkably, siPDK1-transfected SKOV-3 and shPDK1-transfected A2780CP cells displayed attenuated α5 and β1 integrin mRNA (Fig. 2c, upper; Supplementary Fig. 2A) and protein expression (Fig. 2c, lower), whereas ectopic expression of PDK1 in cells exerted the opposite effects. Moreover, transfection with siRNAs against α5 or β1 integrin (Supplementary Fig. 2B) blocked PDK1-mediated cell adhesion to fibronectin and mesothelial cells (Fig. 2b, lower).

### PDK1 induces cell migration and invasion and is involved in anchorage-independent growth

**Fig. 3:**
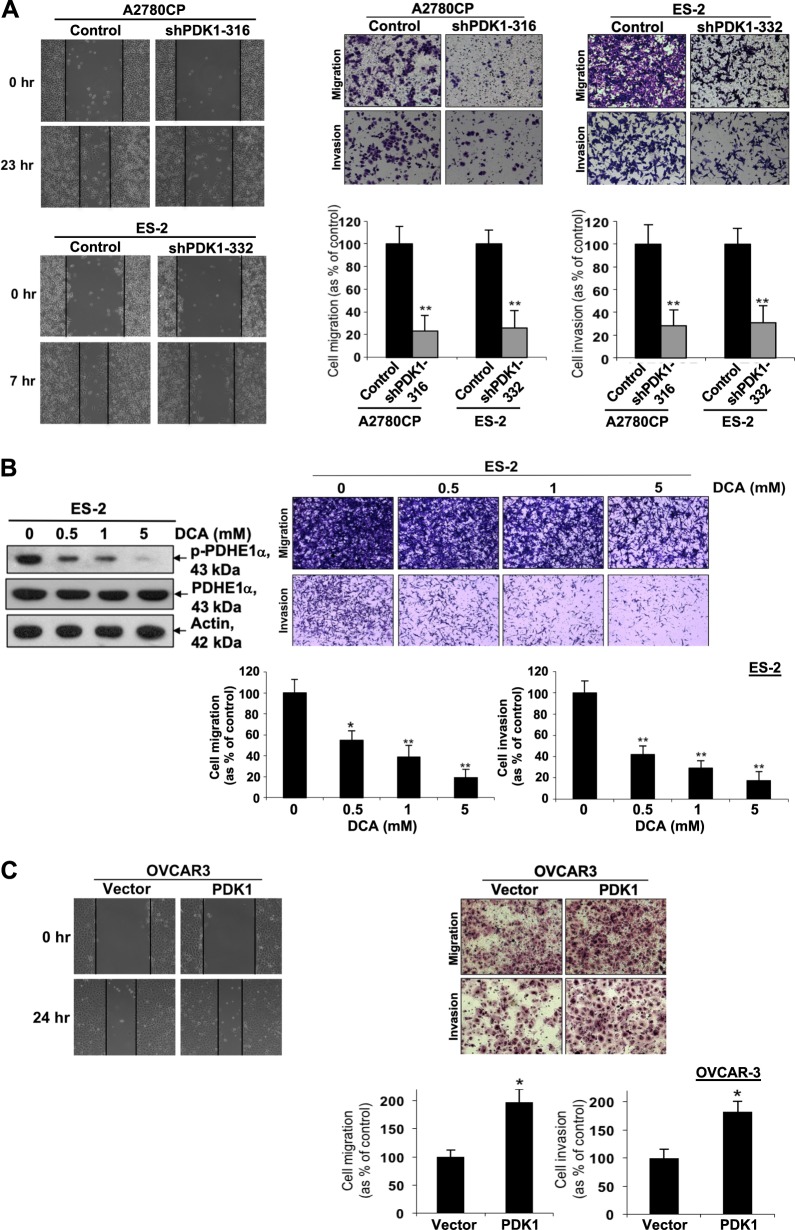
PDK1 contributes to cell migration and invasion. **a** Left: Wound-healing assay with A2780CP and ES-2 cells (control and stable knockdown of PDK1). Cells seeded in six-well plates for 24 h were wounded with a sterile pipette tip, followed by the addition of fresh culture medium. Images were obtained at the same wound position at time 0 and 7 (for ES-2) or 23 h (for A2780CP). Right: Cells were plated in the medium on the upper side of a transwell chamber and allowed to migrate through an 8-µm pore size membrane or invade a Matrigel-coated membrane toward lower chamber with medium plus 10% FBS (as a chemoattractant). After 12–48 h, cells on the upper compartment of the membrane were removed, and migrated or invaded cells were fixed, stained, and counted. Migration or invasion of A2780CP (shPDK1-316) and ES-2 cells (shPDK1-332) with stable knockdown of PDK1 presented as a percentage of controls; Bars: mean ± SD of three experiments; **P < 0.005, Mann–Whitney test. Representative images of migrating or invading A2780CP and ES-2 cells (upper). **b** Left: p-PDHE1α and PDHE1α protein expression in DCA-treated and control ES-2 cells determined via immunoblot analysis Right: Migration or invasion of DCA-treated and control ES-2 cells presented as a percentage of controls; Bars: mean ± SD of three experiments; **P < 0.005, Mann–Whitney test. Representative images of migrating or invading ES-2 cells (upper). **c** Left: Wound-healing assay performed on control and PDK1-overexpressing OVCAR-3 cells. Right: In vitro migration and invasion assays performed using control and PDK1-overexpressing OVCAR-3 cells. Representative images of migrating or invading OVCAR-3 cells (upper). Cell migration or invasion presented as a percentage of controls; Bars: mean ± SD of three experiments; *P < 0.05, **P < 0.005, Mann–Whitney test.

After disseminated primary ovarian cancer cells home to the omentum or peritoneum and interact with mesothelial cells, ovarian cancer cells migrate and invade through the mesothelial cell layer into the submesothelial extracellular matrix (ECM) to establish metastasis^26^. Data from the wound-healing assay revealed a slower migration rate of A2780CP and ES-2 cells following shPDK1-mediated knockdown (Fig. 3a, left). Moreover, both cell migration and invasion abilities were decreased in transiently silenced SKOV-3 and A2780CP as well as cell lines (A2780CP and ES-2) with stable silencing of PDK1, compared with control cells (Fig. 3a, right; Supplementary Fig. 3A, B). We also investigated the effects of DCA, a PDK inhibitor, on A2780CP and ES-2 cells. DCA, a pyruvate analog, binds the N-terminal region of PDK, leading to PDK inhibition. The compound has been used to treat mitochondrial diseases with low toxicity profiles and has been tested in a Phase I trial in patients with recurrent brain tumors^11,27^. DCA suppressed p-PDHE1 expression in a dose-dependent manner (Fig. 3b; Supplementary Fig. 3C, left), indicating that DCA inhibits PDKs, thus blocking PDH phosphorylation. Moreover, DCA retarded the migration and invasion abilities of A2780CP and ES-2 cells in a dose-dependent manner (Fig. 3b, right; Supplementary Fig. 3C, right). Conversely, cell migration and invasion abilities were increased in PDK1-overexpressing OVCAR-3 cells, compared with control group (Fig. 3c). Since our invasion chambers were coated with Matrigel which do not require matrix metallopeptidases (MMP) activation for cell penetration^28^, our invasion assay did not provide any data supporting MMP expression or activation. We further evaluated the effects of PDK1 on ovarian cancer cell growth. PDK1-depleted cells did not display alterations in cell proliferation, as determined using XTT (Supplementary Fig. 4A) and cell counting methods (Supplementary Fig. 4B). However, fewer colonies were formed in PDK1-depleted A2780CP cells on soft agar medium (Supplementary Fig. 4C), suggesting that PDK1 can induce anchorage-independent growth.

### IL-8 is a downstream target of PDK1 that mediates migration, invasion, and angiogenesis

**Fig. 4:**
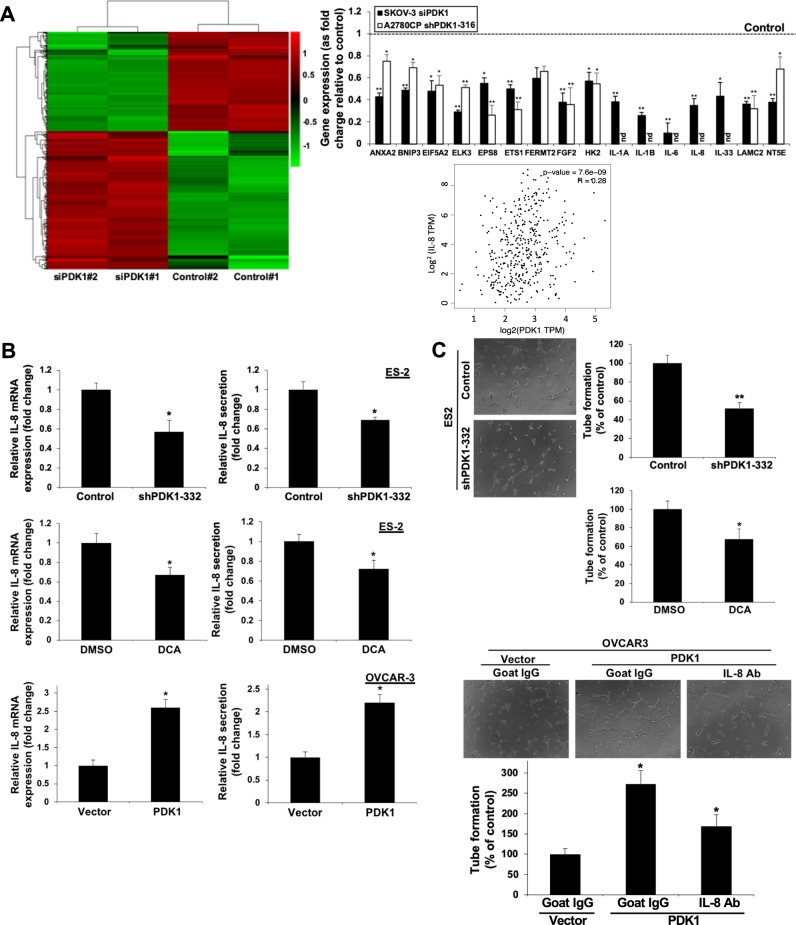
PDK1 drives metastasis and angiogenesis through IL-8. **a** Left: Hierarchical clustering heatmap of differentially expressed genes between control and siPDK1-transfected SKOV-3 cells (log_2_ fold change ≥1 cutoff). Upper right: mRNA expression of 16 metastasis-related genes, calculated as fold change relative to control in PDK1-silenced SKOV-3 (siPDK1) and A2780CP (shPDK1-316) cells using qPCR; Bars: mean ± SD of 3 experiments; *P < 0.05; **P < 0.005; nd non-detectable, Mann–Whitney test. Lower right: Correlation between IL-8 and PDK1 in ovarian cancer in TGCA database cohorts determined using the GEPIA tool. **b** IL-8 mRNA expression and secretion in ES-2 cells with stable knockdown of PDK1 (shPDK-332) (upper), 1 mM DCA treatment (middle), and PDK1-overexpressing OVCAR-3 cells (lower) via qPCR (left) and ELISA (right), presented as fold change relative to control; bars: mean ± SD of three experiments; *P < 0.05, Mann–Whitney test. **c** Human umbilical vein endothelial cells (HUVEC) in supplemented Medium 200 were plated on Geltrex® Matrix-coated 96-well plates. Conditioned medium was added to each well. After 4–8 h, the number of tubes formed was imaged using SPOT imaging software and counted in three random fields under a ×20 objective lens. Capillary tube formation by HUVECs treated with conditioned medium from ES-2 with stable knockdown of PDK1 (shPDK-332) (upper), 1 mM DCA treatment (middle), and PDK1-overexpressing OVCAR-3 cells in the presence or absence of neutralizing antibodies to IL-8 (lower), presented as a percentage of controls; bars: mean ± SD of three experiments; *P < 0.05, **P < 0.005, Mann–Whitney test.

Reduced lactate levels after knockdown of PDK1 may contribute to effects on cell migration and invasion, since lactate is known to enhance tumor cell motility in head-and-neck carcinoma cell lines^29^ and ECM breakdown^30^. It is still worthy to investigate the molecular pathways through which PDK1 mediates cell migration and invasion. To this end, RNA sequencing (RNA-Seq) analysis was employed to compare the gene expression profiles of SKOV-3 cells with and without PDK1 depletion (control versus siPDK1). Based on log_2_ fold change ≥1 and adjusted *p*-value ≤0.05 criteria, we identified 528 deregulated genes (221 downregulated and 307 upregulated). A hierarchical clustering heatmap was generated to demonstrate the differential expression patterns between control and siPDK1-transfected SKOV-3 cells (Fig. 4a), and Metascape analysis applied to assess the functions of differentially expressed genes (DEGs). Genes downregulated in response to siPDK1 treatment were associated with “vasculature development includes cell migration”, “signaling by interleukins”, and “hypoxia induced factor (HIF) 1 pathway” (Supplementary Fig. 5A), which are functions involved in cancer metastasis. On the other hand, genes upregulated in the presence of siPDK1 were associated with “influenza viral RNA transcription and replication”, “heart field specification”, and “transcriptional regulation by small RNAs” (Supplementary Fig. 5B). Gene set enrichment analysis (GSEA) was additionally performed to identify differentially regulated gene sets. Similar to Metascape analysis, “epithelial-mesenchymal transition (EMT) includes wound healing, fibrosis, and metastasis”, and “hypoxia” were within the top three significantly enriched gene sets identified (Supplementary Table 3), further supporting the theory that PDK1 drives ovarian cancer metastasis. Among those gene sets, 16 genes were linked to metastasis through PubMed literature searches, including ANXA2, BNIP3, EIF5A2, ELK3, EPS8, ETS1, FGF2, FERMT2, HK2, IL-1A, 1L-1B, IL-6, IL-8, IL-33, LAMC2, and NT5E (Supplementary Fig. 5C). We confirmed significant downregulation of these 16 genes in PDK1-depleted SKOV-3 and A2780CP cells via qPCR, except that all five interleukins were undetectable in A2780CP cells (Fig. 4a, upper right; Supplementary Fig. 5D). In addition, consistent with qPCR analyses (Fig. 2c), RNA-seq analysis also revealed decrease of α5 (0.68-fold vs. control; log_2_ fold change = 0.55; *p*-value <0.05) and β1 (0.65-fold vs. control; log_2_ fold change = 0.62; *p*-value <0.05) integrin mRNA expression in SKOV-3 cells.

Next, we examined the clinical correlation between these selected 16 DEGs and PDK1 expression using GEPIA. A significant correlation between BNIP3, EIF5A2, ELK3, EPS8, FERMT2, HK2, IL-1A, IL-6 and IL-8, and PDK1 expression (*P* < 0.05) in ovarian cancer clinical samples was found (Fig. 4a, lower right; Supplementary Fig. 6).

shPDK1 (Fig. 4b, upper; Supplementary Fig. 7A) and DCA treatment (Fig. 4b, middle) of ES-2 cells induced a significant decrease in IL-8 mRNA and protein concentrations, as observed with qPCR and ELISA, respectively, along with reduced capacity to mediate angiogenesis assessed via the endothelial cell tube-formation assay (Fig. 4c, upper and middle; Supplementary Fig. 7B). IL-8 treatment enhanced cell migration and invasion of ES-2 cells (Supplementary Fig. 7C), supporting its involvement in metastasis, consistent with previous findings. Conversely, ectopic expression of PDK1 significantly promoted IL-8 mRNA and protein expression (Fig. 4b, lower). Notably, PDK1-mediated tube formation was blocked upon treatment of CM with neutralizing antibodies against IL-8 (Fig. 4c, lower). In addition to angiogenesis, “hypoxia-induced factor (HIF) 1 pathway”/“hypoxia” gene sets were enriched among those downregulated in the siPDK1-transfected group (Supplementary Fig. 5A and Supplementary Table 3). This finding is in keeping with an earlier report that knockdown of PDK1 suppress HIF1A stabilization in HNSC^13^. Given that PDK1 can be induced by HIF1^11,12^, the link between PDK1 and HIF1 in ovarian cancer will be further investigated in future experiments.

### PDK1 induces IL-8 expression, cell migration, invasion, and angiogenesis via JNK signaling

**Fig. 5:**
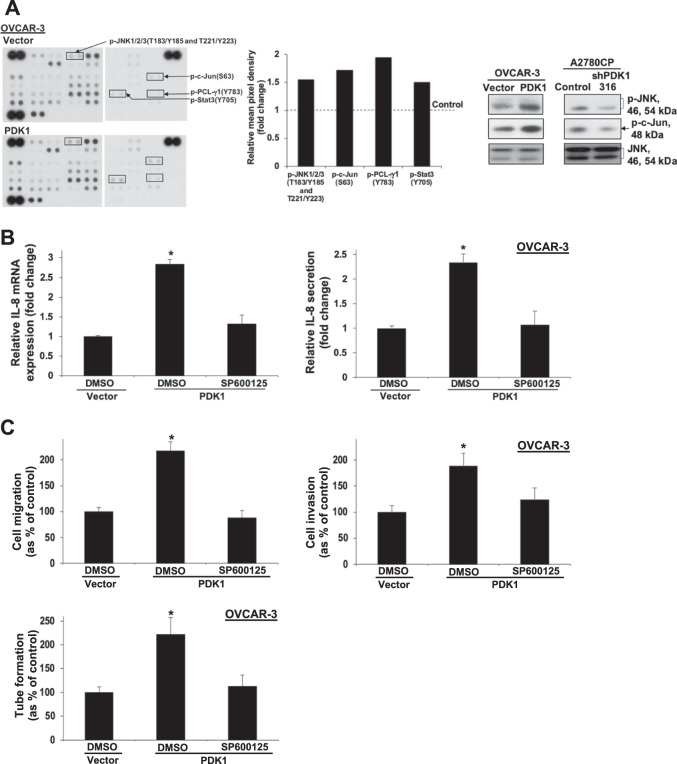
Phospho-kinase array profiling analysis showing involvement of JNK in PDK1-induced IL-8 expression, metastasis, and angiogenesis. **a** Left: Immunoblot analyses of the Proteome Profiler Human Phospho-Kinase Array comparing OVCAR-3 cells with and without PDK1 overexpression. Middle: The spot pixel density on the array was quantified using ImageJ software. The spot targets with >1.5-fold changes between the two groups were recorded. The mean pixel density of p-JNK1/2/3 (T183/Y185 and T221/Y223), p-c-Jun (S63), p-PCL-γ1 (Y783), and p-Stat3 (Y705) in OVCAR-3 cells with stable PDK1 overexpression. Right: Immunoblot analyses of p-JNK, p-c-Jun, and JNK in PDK1-overexpressing OVCAR-3 and PDK1-depleted A2780CP (shPDK1-316) cells. **b** IL-8 mRNA expression and protein secretion in control or PDK1-overexpressing OVCAR-3 cells treated with DMSO (vehicle) or the JNK inhibitor, SP600125, determined via qPCR (left) and ELISA (right), presented as fold change relative to controls; bars: mean ± SD of three experiments; *P < 0.05; Mann–Whitney test. **c** Upper: In vitro migration and invasion assays with control or PDK1-overexpressing OVCAR-3 cells treated with DMSO or SP600125. Cell migration and invasion are presented as a percentage of controls; bars: mean ± SD of three experiments; *P < 0.05; Mann–Whitney test. Lower: Capillary tube formation by HUVECs treated with conditioned medium from control or PDK1-overexpressing OVCAR-3 cells subjected to DMSO or SP600125 treatment.

To clarify the mechanistic pathways by which PDK1 regulates ovarian cancer metastasis, we screened lysates from OVCAR-3 cells with or without stable PDK1 overexpression. ImageJ analysis was applied to quantify the average signal (pixel density) of the pair of duplicate spots representing each phosphorylated kinase protein on the array. The mean pixel density of p-JNK1/2/3 (T183/Y185 and T221/Y223), p-c-Jun (S63), p-PCL-γ1 (Y783), and p-Stat3 (Y705) showed >1.5-fold increase in OVCAR-3 cells with stable PDK1 overexpression (Fig. 5a, left and middle). Increased levels of p-JNK and p-c-Jun were confirmed via western blot (Fig. 5a, right). We additionally observed a decrease in p-JNK and p-c-Jun levels in PDK1-depleted A2780CP cells. Subsequent GSEA revealed that DEGs downregulated in response to siPDK1 are associated with JNK downstream signaling gene sets, including activator protein-1 (AP-1), Elk1, and nuclear factor of activated T cell (NFAT) (Supplementary Table 4)^31^. Remarkably, the JNK pathway is one of mechanisms promoting IL-8 expression.

To further validate the role of JNK and examine the possible association between JNK and IL-8, PDK1-overexpressing OVCAR-3 cells were treated with SP600125 (10 μM), a JNK inhibitor. qPCR and ELISA experiments revealed blockage of IL-8 mRNA and protein expression, respectively, by SP600125 in OVCAR-3 cells with PDK1 overexpression (Fig. 5b). In addition, SP600125 inhibited PDK1-driven cell migration, invasion, and angiogenesis (Fig. 5c), clearly suggesting that metastasis induced by PDK1 via upregulation of IL-8 is JNK-dependent.

### PDK1 enhances tumor growth and dissemination in nude mice

**Fig. 6:**
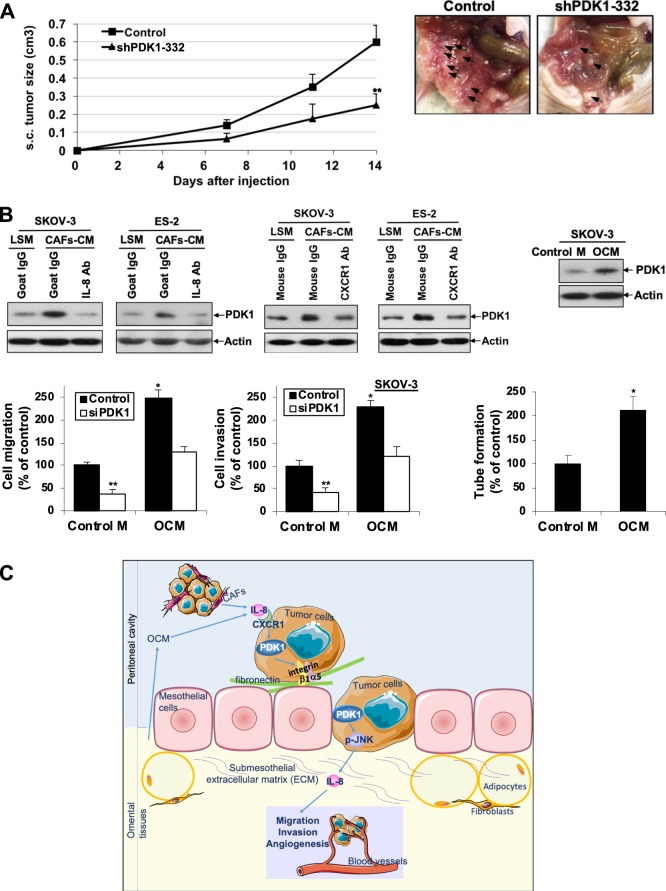
PDK1 abrogation inhibits tumor growth and dissemination in nude mice. CAF-CM derived from ovarian cancer and OCM induced PDK1 expression in ovarian cancer cells through IL-8/CXCR1. **a** Left: BALB/c female nude mice were injected s.c. (five mice/group) with ES-2 cells stably depleted of PDK1 (2 × 10^6^). Perpendicular tumor diameters were measured on days 7, 11, and 14, and tumor volumes calculated. Growth rates of s.c. tumors formed in mice inoculated with shPDK1-332 ES-2 or control cells; bars: mean ± SD; **P < 0.005, Mann–Whitney test. Right: BALB/c female nude mice were injected i.p. (seven mice/group) with ES-2 cells stably depleted of PDK1 (2 × 10^6^). Mice were killed 14 days after cell injection. Tumor dissemination was observed. Representative views of the abdominal cavity of mice inoculated i.p. with shPDK1 ES-2 or control cells. Arrows, tumors. **b** Upper left: Immunoblot analysis of PDK1 expression in serum-starved SKOV-3 and ES-2 cells treated with CAF-CM in the presence or absence of neutralizing antibodies to IL-8, CXCR1 or corresponding control IgG for 48 h. LSM: low serum complete medium (control medium for CAF-CM). Upper right: Immunoblot analysis of PDK1 expression in serum-starved SKOV-3 cells treated with OCM for 48 h. Control M: DMEM/F12 without phenol red (control medium for OCM). Lower left: Cell migration and invasion of siPDK1 SKOV-3 cells with or without OCM treatment presented as a percentage of controls; bars: mean ± SD of three experiments; *P < 0.05; **P < 0.005; Mann–Whitney test. Lower right: Capillary tube formation by HUVECs treated with or without OCM presented as a percentage of controls; bars: mean ± SD of three experiments; *P < 0.05; Mann–Whitney test. **c** Schematic illustration showing that CAFs and OCM in the tumor microenvironment induce PDK1 through IL-8/CXCR1. PDK1, in turn, promotes cell adhesion, migration, invasion, and angiogenesis in ovarian cancer cells, contributing to metastasis. The underlying mechanisms involve modulation of α5β1 integrin expression and activation of the JNK/IL-8 signaling pathway.

To establish the effects of PDK1 depletion on in vivo tumor growth and dissemination, shPDK1 ES-2 and control cells were inoculated subcutaneously (s.c.; *n* = 5) or intraperitoneally (i.p.; *n* = 7) into nude mice. PDK1 knockdown resulted in significant reduction of tumor growth (Fig. 6a, left). Upon examination of the peritoneal cavity of mice at 14 days after i.p. inoculation, control mice displayed extensive abdominal dissemination predominantly at the mesentery, whereas only focal nodules were detected in mesentery of mice with PDK1-depleted cells (Fig. 6a, right). At the time of killing, tumors were excised and weighed, the total i.p. tumor weight in the shPDK1 ES-2 cell-injected mice was significantly lower than that in the control mice (Supplementary Fig. 8A). Excised tumors were snap-frozen for RNA extraction. We found that IL-8 mRNA expression in i.p. tumors with PDK1-depleted cells was significantly decreased compared with that in control tumors by qPCR (Supplementary Fig. 8B).

### CAF-CM and OCM upregulate PDK1 expression in ovarian cancer cells. OCM contributes to ovarian cancer metastasis via PDK1

CAF is a major component of the tumor microenvironment, which contributes to ovarian cancer progression and facilitates dissemination through secretion of chemokines and the extracellular matrix^32,33^. To elucidate the mechanisms governing PDK1 upregulation in ovarian cancer cells, we first attempted to determine whether conditioned medium from CAFs regulates PDK1 expression in SKOV-3 and ES-2 cells. Intriguingly, PDK1 expression was enhanced upon CAF-CM treatment (Fig. 6b, upper left). Given that IL-8 is upregulated in CAF-CM from ovarian cancers^34^, we further found that CAF-CM-induced PDK1 expression is blocked upon treatment with neutralizing IL-8 and CXCR1 antibodies (Fig. 6b, upper left). Since IL-8 is a major chemokine in omentum^35^, the major site of ovarian cancer metastasis^2,3^, we also examined PDK1 expression upon OCM treatment in SKOV-3 cells, which consistently showed upregulation (Fig. 6b, upper right). We further observed that OCM promotes ovarian cancer cell migration and invasion (Fig. 6b, lower left), along with increased capacity to mediate angiogenesis (Fig. 6b, lower right). In addition, knockdown of PDK1 blocked OCM-mediated migration and invasion (Fig. 6b, lower left), suggesting that metastasis induced by OCM is PDK1-dependent.

## Discussion

In this study, we demonstrated upregulation of the gatekeeping enzyme, PDK1, in ovarian cancer patients, with significantly higher expression in metastatic foci. Our results additionally showed that PDK1 phosphorylates/inactivates PDHE1 leading to increased lactate production in ovarian cancer cells. Higher glucose consumption, pyruvate uptake, and lactate production have been documented in invasive ovarian cancer cells^36^. Previous reports have highlighted an increase in lactate levels in both primary and metastatic ovarian cancer, compared with normal ovarian tissue^20^. Here, we have provided evidence that PDK1 is involved in this metabolic switch. Importantly, higher PDK1 expression was correlated with shorter overall and disease-free survival, and PDK1 is an independent prognostic factor for overall survival, suggesting that PDK1 is a significant prognostic marker in ovarian cancer. The relatively low level of PDK1 in OVCAR-3 cells may be due to different genetic backgrounds of different cell lines. Such finding can be taken as evidence that an upregulation of PDK1 may not be observed in all ovarian cancers. The cell of origin for the various histotypes of ovarian cancer is still a challenging issue. Numerous studies have revealed the fallopian tube epithelium as the site of origin for most high-grade serous ovarian cancers and that the site of clear cell and endometroid ovarian cancers may originate from sites other than the ovarian surface epithelium. Thus, the use of HOSE may not provide a complete comparison of PDK1 expression in malignant versus malignant cells.

Significantly higher PDK1 expression in metastatic foci was detected, indicative of its potential contribution to ovarian cancer metastasis. Ovarian cancer preferentially disseminates in the peritoneal cavity. Adhesion of ovarian cancer cells carried by peritoneal fluid to the mesothelium cells covering the peritoneal cavity is considered a prerequisite for metastasis^2,3^. Mesothelial cells have also been shown to promote ovarian cancer cell adhesion^37^ and metastasis^38^. Fibronectin, collagen, and laminin are extracellular matrix proteins present in both ascites and mesothelium^3^. A recent study revealed that fibronectin secreted by mesothelial cells contributes to early ovarian cancer metastasis^38^. In our experiments, knockdown of PDK1 reduced ovarian cancer cell adhesion to fibronectin and mesothelial cells along with downregulation of α5 integrin and β1 integrin, whereas ectopic overexpression exerted the opposite effects, supporting a role of PDK1 in the early steps of metastasis.

Cell-matrix adhesion is mediated by transmembrane receptor integrins composed of α- and β-subunits. α5β1 integrin is a fibronectin receptor. After ligand binding, integrins activate signaling pathways that, in turn, regulate various cellular processes^3,25^. Both α5 and β1 integrin are prognostic markers in ovarian cancer^39^. Inhibition or blocking of these integrins via the siRNA approach or antibody treatment has been shown to reduce ovarian cancer cell adhesion to fibronectin and mesothelial cells^25,37,38^. In previous studies, α5β1 integrin blockade attenuated the invasive ability of ovarian cancer cells^38^ and treatment with α5 or β1 integrin antibodies or manipulation of β1 integrin expression inhibited ovarian cancer dissemination in vivo^25,38^. In our investigation, transient knockdown of α5 or β1 integrin abolished PDK1-mediated ovarian cancer cell adhesion to fibronectin and mesothelial cells, clearly suggesting that cell adhesion to fibronectin and mesothelial cells induced by PDK1 is α5β1 integrin-dependent.

In addition to cell adhesion, our functional studies involving manipulation of PDK1 expression in ovarian cancer cells demonstrated roles in promoting cell migration, invasion, and angiogenesis, which are critical steps for metastasis, especially in the peritoneal microenvironment^2,3^. RNA sequencing analysis comparing SKOV-3 cells with and without knockdown of PDK1 led to the identification of DEGs related to migration and angiogenesis, and consequently metastasis. The clinical relevance of PDK1 in metastasis was further supported by its correlations with DEGs associated with metastasis in TCGA data sets of ovarian cancer patients. Moreover, treatment with the PDK inhibitor, DCA, impaired cell migration, invasion and angiogenesis. In vivo, PDK1 knockdown attenuated tumor dissemination in nude mice. Taken together, our results support a crucial role of PDK1 in ovarian cancer metastasis. Phosphoglycerate kinase (PGK), another glycolytic enzyme, has been shown to promote peritoneal metastasis of gastric cancer^40^. In addition, mitochondrial PGK functions as a protein kinase to phosphorylate PDK1, regulating the Warburg effect and promoting brain tumorigeneses^40^. Weather PDK1 can be phosphorylated by PGK1, leading to ovarian cancer tumorigeneses and metastasis will be examined in future studies.

Currently, there is an active search for more target agents involved in angiogenesis^41^. Here, we have demonstrated that PDK1 induces IL-8, an angiogenic CXC chemokine highly expressed in ovarian cancers and ascites^42,43^. High IL-8 expression in ovarian cancer appears to be associated with advanced stage and poor prognosis^42^. Together with previous studies, our current experiments demonstrate promoting effects of IL-8 on ovarian cancer cell migration and invasion^42^. IL-8 binds to its receptors, CXCR1 and CXCR2, expressed in endothelial cells. This binding in turn mediates angiogenic effects and strongly promotes tumor metastasis^43^. Gene silencing of IL-8 in ovarian cancer has also been shown to inhibit tumor growth and retard metastasis dissemination in vivo^42^. Data from our study indicate that PDK1 mediates cell migration, invasion, and angiogenesis, and thus metastasis via upregulation of IL-8. Moreover, the lack of IL-8 in A2780CP cells suggested that other downstream target(s) would be involved in PDK1-mediated effects in ovarian cancer.

Activation of EGFR by PDK1 has been shown to play a role in sustaining malignant progression in glioblastoma^44^. Here, we have reported a mechanism involving PDK1-mediated activation of the JNK/IL-8 downstream pathway. The identified link between PDK1 and IL-8 supports that the dual targeting of PDK1 (for instance, with DCA) and IL-8 (for instance, with small-molecule antagonists and humanized monoclonal antibodies) should be further studied in ovarian or other cancers as possible novel therapeutic approaches. In addition, tumor suppressor cylindromatosis has been found to regulate melanoma metastasis through the β1-integrin/JNK signaling pathway^45^. On the other hand, β1-integrin-induced IL-8 production through the RAC1/P38 MAPK signaling pathway was documented in human natural killer cells^46^. Future studies will explore whether α5β1 integrin is involved in JNK/IL-8 activation leading to ovarian cancer metastasis.

Knockdown of PDK1 did not affect ovarian cancer cell proliferation, but retarded anchorage-independent growth. In vivo, PDK1 depletion impeded tumor growth in nude mice. This finding is not unexpected, since in a previous study showing that in vitro PDK1 knockdown impedes in vivo HNSC tumor growth, no differences in cell proliferation but a reduction in anchorage-independent growth was observed^13^.

Emerging evidence suggests that the tumor microenvironment is indispensable in ovarian cancer progression and metastasis, although the underlying mechanisms remain elusive^33,47^. In ovarian tumors, fibroblasts in the stroma interacting with ovarian cancer cells are transformed into CAFs, which constitute more than half the tumor microenvironment^32,33^. CAFs have been shown to promote ovarian cancer cell growth, adhesion, invasion and metastasis by secreting chemokines and ECM, facilitating dissemination^32^. A recent study showed that CAFs in peritoneum derived from mesothelial cells enhanced ovarian cancer cell metastasis^48^. Intriguingly, in ovarian cancer, IL-8 was identified as one of the three highest upregulated chemokines in CAFs^34^. In this study, treatment with conditioned medium from CAFs isolated from ovarian tumor led to upregulation of PDK1 expression. This induction was impeded by blockage of IL-8 and CXCR1, suggesting that CAFs secrete chemokines, such as IL-8, which, in turn, enhance PDK1 expression in ovarian cancer cells through CXCR1. Other than tumor cells and CAFs, IL-8 is secreted by numerous cell types, such as mesothelial cells, endothelial cells, and monocytes^43^. IL-8 secreted by omental adipocytes has also been shown to promote adhesion, migration, and invasion of ovarian cells to omentum through CXCR1^35^. Our results demonstrate that OCM induces PDK1, which, in turn, contributes to ovarian cancer migration, invasion, and angiogenesis, thus metastasis.

In conclusion, we detected significant overexpression of PDK1 in ovarian cancer. High PDK1 expression was associated with cancer metastasis and poor patient outcomes. Proteins secreted by CAFs and omental tissues, such as IL-8, in the tumor microenvironment appeared to contribute to PDK1 upregulation and its metastasis functions. In addition, PDK1 played regulatory roles in the metabolic switch and promoted cell adhesion, migration, invasion, angiogenesis, and anchorage-independent growth in ovarian cancer cells, leading to metastasis. The underlying mechanisms involved regulation of α5β1 integrin and JNK/IL-8 signaling pathways (Fig. 6c). Improved understanding of the involvement of PDK1 in metastasis should facilitate its effective application as a therapeutic molecular target, either alone or in combination with other molecular targets.

## Materials and methods

### Clinical samples and cell lines

Paraffin-embedded samples of 102 patients with ovarian cancer were retrieved from the Department of Pathology, Queen Mary Hospital, University of Hong Kong. All patients underwent surgery, with 77 further receiving chemotherapy, including platinum/paclitaxel. The mean follow-up period was 63 months (range, 5–209 months). 28 matched metastatic foci from 17 primary tumors were also retrieved. Twenty paired frozen blocks of ovarian cancer samples and their corresponding normal counterparts, including fallopian tubes and/or contralateral ovaries, were collected for mRNA expression analysis. Approval for sample usage was obtained by the Institutional Ethical Review Board (UW 16-107). Informed consent was obtained from all subjects. Immortalized ovarian epithelial cell lines and ovarian cancer cell lines were cultured as described previously^49,50^.

### Transient and stable silencing of PDK1 and DCA treatment

For transient silencing, siRNA specifically targeting PDK1 (sc-36203) and control siRNA (Santa Cruz Biotechnology, Inc., Santa Cruz, CA) were introduced into SKOV-3 and A2780CP cells using SilentFect (Bio-Rad Laboratories, Hercules, CA) for 48 h before cell counting and plating for subsequent assays^49,50^. The time and dose were optimized to ensure a maximal decrease. For stable silencing, A2780CP and ES-2 cells were transfected with SureSilencing shRNA plasmids against human PDK1 and a negative control (Qiagen, Valencia, CA) using Lipofectamine 3000 (Invitrogen, San Diego, CA), and stable clones were selected with puromycin (1.5 µg/ml)^49,50^. A2780CP and ES-2 cells were plated 24 h before treatment with the PDK inhibitor, dichloroacetate (DCA) (Sigma-Aldrich, St. Louis, MO) or vehicle (DMSO)^49–51^.

### Transient and stable overexpression of PDK1 and treatment with siRNAs

pCMV6-DDK-PDK1 and pCMV6-DDK (control vector) purchased from Origene (Rockville, MD) were transfected into OVCAR-3 using Lipofectamine 3000. After 72 h, transiently transfected cells were counted and plated for subsequent assays. For overexpression, stable clones were selected using G418 (500 µg/ml). OVCAR-3 cells stably overexpressing PDK1 were plated and transfected with siRNAs specific for α5 integrin (sc-29372), β1 integrin (sc-35674), or control (sc-37007) (Santa Cruz) for 48 h before cell counting, labeling, and plating for functional assays^49,50^.

### General methods

Immunohistochemical analyses, real-time PCR (qPCR), and immunoblotting were performed as described previously^49,50^. Immunoreactivity was assessed by multiplying intensity by the percentage of stained cells. Intensity was scored as 0 (negative), 1 (faint), 2 (moderate), or 3 (strong). The percentage was rated as 0 (<5%), 1 (5–25%), 2 (26–50%), 3 (51–75%), or 4 (>75%). Scorers were blinded to the treatment conditions. Primary antibodies and primer sequences are listed in Supplementary Tables 5 and 6, respectively. The human phospho-kinase array kit (ARY003B) was obtained from R&D Systems (Minneapolis, MN).

### ELISA and lactate assays

Media from cultured cells were collected and used for measuring IL-8 and lactate levels with Human IL-8 (Biolegend, San Diego, CA) ELISA kit^42^ and Lactate Colorimetric Assay Kit II (BioVision)^17^.

### Adhesion assay

96-well plates were coated with fibronectin (10 μg/ml), human mesothelial cells MeT-5A (ATCC, Manassas, VA), or primary human adult omentum-derived mesothelial cells (Zen-Bio Inc., Research Triangle Park, NC). Ovarian cancer cells (3 × 10^4^ cells/well) labeled with 5 μM calcein-AM (Invitrogen) were added on top. After 45 min, total fluorescence was measured at 485 nm (excitation) and 535 nm (emission). Fluorescence was measured after washing off nonadherent cells five times. The percentage of bound cells was calculated based on fluorescence after washing, compared with total fluorescence^25^.

### Functional assays and in vivo studies

Wound healing, in vitro migration and invasion assays, XTT, cell counting, soft agar assays, and in vivo studies were performed as described previously^49,50^. In vivo experiments were performed following the Animals (Control of Experiments) Ordinance (Hong Kong) and Institute’s guidance on animal experiments. For OCM (Zen-Bio Inc.) treatment, the lower side of transwell chambers lower chambers were filled with phenol red-free DMEM/F12 plus 1% FBS or OCM plus 1% FBS as a chemoattractant.

### Tube-formation assay

Media from cultured cells were collected, filtered, and stored at −80 °C as conditioned medium. An Angiogenesis Starter Kit (Life Technology) was used to determine the formation of three-dimensional vessels according to the manufacturer’s protocol. Briefly, human umbilical vein endothelial cells (HUVEC) in supplemented Medium 200 (8000 cells/50 μl/well) were plated on Geltrex® Matrix-coated (100 μL per cm^2^) 96-well plates and conditioned medium/OCM (200 μl) added to each well. After 4–8 h, the number of tubes formed was imaged using SPOT imaging software and counted in three random fields under a ×20 objective lens. A connection between two cells was counted as one capillary tube formed.

### RNA sequencing and analysis

RNA preparation, library construction, and sequencing with the BGISEQ-500 platform were performed at Beijing Genomics Institute (BGI, Shenzhen, China). Gene expression levels were quantified using RNA-Seq by Expectation Maximization (RSEM)^52^. The DEseq2 method was applied to detect DEG between groups (*n* = 2) using Benjamini–Hochberg (BH) method for correcting for multiple comparisons. Genes were considered significantly differentially expressed at log_2_ fold change ≥1 and adjusted *p*-value ≤0.05. RNA sequencing data sets are available in GEO with accession number GSE141169. Gene Ontology (GO) annotation and pathway enrichment analyses of DEGs were performed using Metascape (http://metascape.org) with the following ontology sources: KEGG Pathway, GO Biological Processes, Reactome Gene Sets, Canonical Pathways and CORUM. Gene set enrichment analysis (GSEA) was additionally performed to identify differentially regulated gene sets, with specific focus on those contained in the molecular signature database (MSigDBv6.1) hallmark and transcription factor target gene lists^53^. GEPIA (http://gepia.cancer-pku.cn/) was further applied to determine the clinical relevance of the DEGs.

### Treatment of cells with conditioned medium in combination with neutralizing antibodies

SKOV-3 or ES-2 cells were serum-starved for 24 h and treated with CAF-CM (Vitro Biopharma, CO) or OCM for 48 h. Serum-starved cells were further treated with neutralizing antibodies against IL-8, CXCR1, or corresponding control IgG (Supplementary Table 5) in combination with CAF-CM for 48 h^49^.

### Statistical analysis

Statistical analyses were performed using SPSS 20 (SPSS Inc., Chicago, IL). For comparing data between and among groups, Mann–Whitney and Kruskal–Wallis rank tests were used, respectively. Kaplan–Meier and log-rank tests were conducted for survival analysis. Multivariate survival was assessed with Cox regression analysis and correlation analysis performed using Spearman’s rho test. *P*-values <0.05 were considered statistically significant.

## Supplementary information

Supp Tables 1-6

Supp Figs 1-8
