# Supplementary material for: PDK1 promotes ovarian cancer metastasis by modulating tumor-mesothelial adhesion, invasion, and angiogenesis via α5β1 integrin and JNK/IL-8 signaling
Source: Oncogenesis. 2020 Feb 18;9(2):24. doi: 10.1038/s41389-020-0209-0 (PMC7028730; doi:10.1038/s41389-020-0209-0)
Supplement: Supplementary file 1 — Supp Tables 1-6 [file 41389_2020_209_MOESM1_ESM.docx]

**Supplementary Table 1.** Correlation of PDK1 histoscore with different diagnostic categories and clinicopathological parameters in ovarian cancer.

| **Characteristics** | **Case (n)** | **PDK1 (histoscore)** | |
| --- | --- | --- | --- |
|  |  | **Mean ± SD** | **p-value** |
| **Diagnostic categories** |  |  |  |
| Benign | 11 | 0±0 |  |
| Carcinomas | 91 | 3.74±3.083 | <0.001* |
| **Stage (FIGO)** |  |  |  |
| I | 37 | 3.76±3.013 |  |
| II | 12 | 3.17±3.689 |  |
| III | 24 | 4.33±3.435 |  |
| IV | 13 | 2.92±2.431 | 0.505* |
| Early (I-II) | 49 | 3.61±3.161 |  |
| Late (III-IV) | 37 | 3.84±3.158 | 0.781† |
| **Histological grade (FIGO)** |  |  |  |
| 1 | 20 | 3.60±2.624 |  |
| 2 | 35 | 3.74±3.266 |  |
| 3 | 33 | 3.61±3.020 | 0.968* |
| Low (1) | 20 | 3.60±2.624 |  |
| High (2-3) | 68 | 3.68±3.126 | 0.806† |
| **Histology** |  |  |  |
| Serous‡ | 35 | 3.77±3.135 |  |
| Clear Cell | 17 | 4.41±3.411 |  |
| Mucinous | 9 | 3.22±2.728 |  |
| Endometrioid | 29 | 3.28±2.877 | 0.726* |
| Serous | 35 | 3.77±3.135 |  |
| Non-serous | 55 | 3.62±3.021 | 0.854† |
| **Chemosensitivity§** |  |  |  |
| Sensitive | 64 | 3.25±2.955 |  |
| Resistant | 13 | 5.08±2.532 | 0.028† |

* Kruskal–Wallis rank test; † Mann-Whitney test; ‡ All serous cancers were high-grade; §Chemosensitive-patients remained disease free more than 6 months after completion of first-line chemotherapy. Those with significant P-values are underlined.

**Supplementary Table 2.** Cox regression analysis for factors affecting overall and disease-free survival.

| **Prognostic factor** | **Overall survival** | | |  | **Disease-free survival** | | | |
| --- | --- | --- | --- | --- | --- | --- | --- | --- |
|  | *P* | Hazard Ratio | 95% Confidence Interval |  | *P* | Hazard Ratio | | 95% Confidence Interval |
| PDHK1 histoscore | 0.048 | 2.245 | 1.007-5.006 |  | 0.162 | | 1.751 | 0.798-3.843 |
| Disease stage | <0.001 | 2.234 | 1.444-3,454 |  | 0.002 | | 1.910 | 1.268-2.878 |
| Grade | 0.014 | 2.320 | 1.187-4.536 |  | 0.087 | | 1.695 | 0.927-3.099 |
| Chemosensitivity | <0.001 | 9.915 | 3.571-27.529 |  | 0.010 | | 3.296 | 1.334-8.143 |

**Supplementary Table 3.** Top three significantly enriched gene sets of differentially expressed genes identified by GSEA.


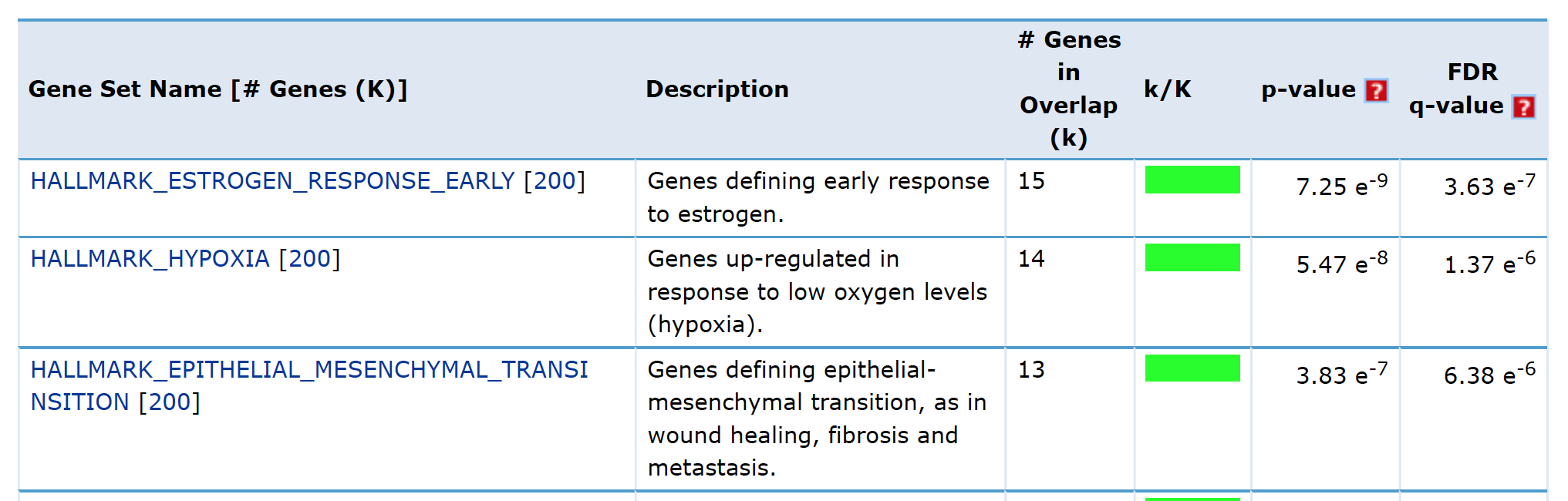


**Supplementary Table 4.** JNK downstream signaling gene sets, including activator protein-1 (AP-1), Elk1 and nuclear factor of activated T cell (NFAT), associated with DEGs downregulated in response to siPDK1.


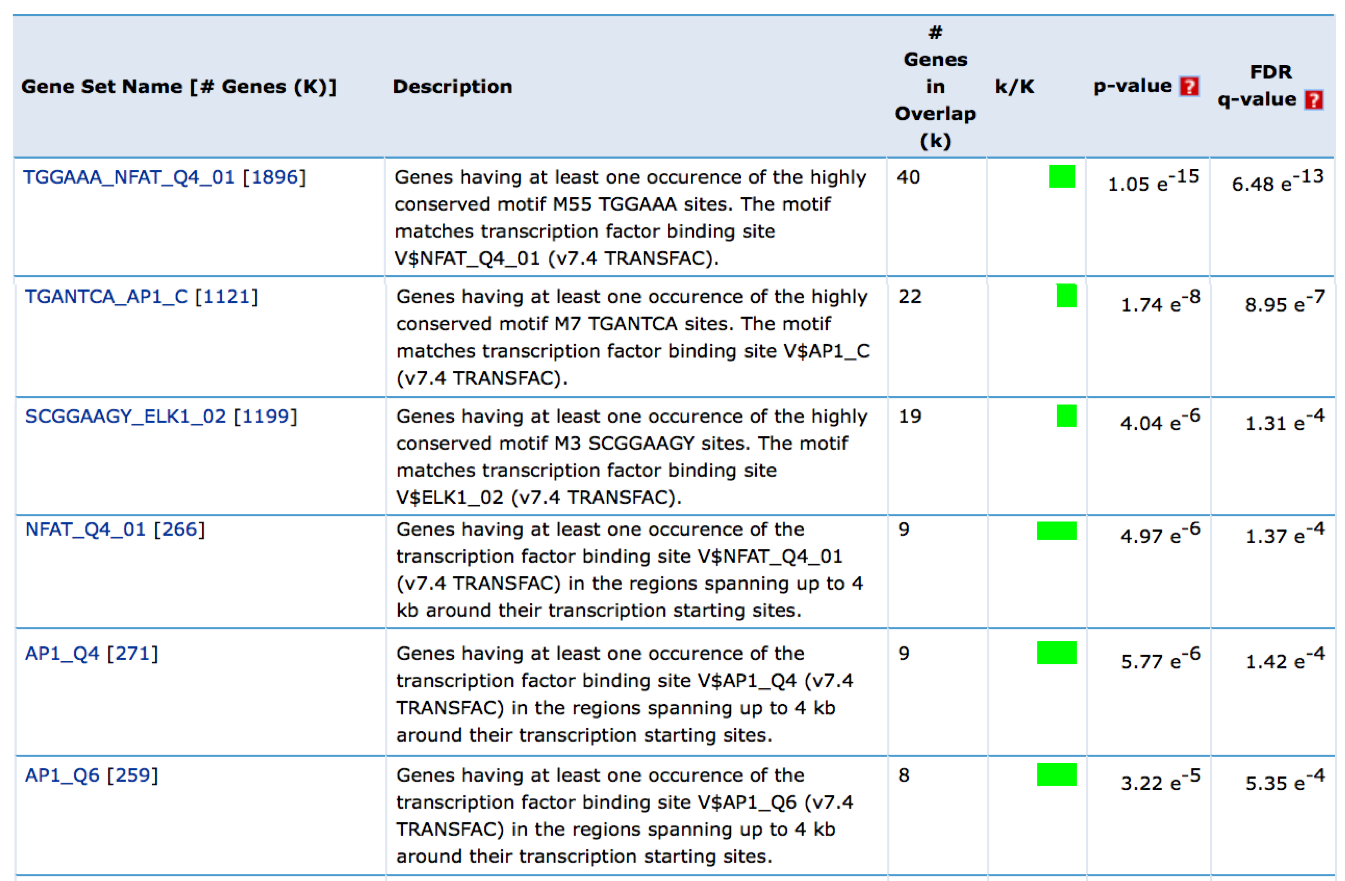


**Supplementary Table 5.** Primary antibodies used for immunohistochemistry, immunoblotting and neutralization.

| **Target Protein** | **Animal source *** | **Catalog #** | **Usage ^**^** | **Working dilution** | **Vendor** |
| --- | --- | --- | --- | --- | --- |
| PDK1 | Rabbit | NBP1-8595 | IHC  IB | 1:100  1:500 | Novus |
| p-PDHE1α | Mouse | NB110-93479 | IB | 1:1000 | Novus |
| PDHE1α | Rabbit | 3205 | IB | 1:1000 | Cell Signaling |
| α5 integrin | Rabbit | 4705 | IB | 1:1000 | Cell Signaling |
| β1 integrin | Goat | sc-6622 | IB | 1:200 | Santa Cruz biotechnology |
| p-JNK | Mouse | sc-6254 | IB | 1:200 | Santa Cruz biotechnology |
| JNK | Rabbit | 9252 | IB | 1:1000 | Cell Signaling |
| p-c-Jun | Rabbit | 3270 | IB | 1:1000 | Cell Signaling |
| IL-8 | Goat | AF-208-NA | N | 2 μg/ml | R&D systems |
| CXCR1 | Mouse | MAB330 | N | 2 μg/ml | R&D systems |
| Actin | Mouse | ab6276 | IB | 1:50000 | Abcan |

* Antibodies prepared in corresponding host animals.

** IB: immunoblotting; IHC: immunohistochemistry; N: neutralization.

**Supplementary Table 6.** Primers used for qPCR.

| Gene | Primer direction | Primer Sequence (5’ to 3’) |
| --- | --- | --- |
| PDK1 | Forward | CCAAGACCTCGTGTTGAGACC |
|  | Reverse | AATACAGCTTCAGGTCTCCTTGG |
| ANXA2 | Forward | GAGCGGGATGCTTTGAACATT |
|  | Reverse | TAGGCGAAGGCAATATCCTGT |
| BNIP3 | Forward | CTGGGTAGAACTGCACTTCAG |
|  | Reverse | GGAGCTACTTCGTCCAGATTCAT |
| EIF5A2 | Forward | TGTCCTTCTACTCACAACATGGA |
|  | Reverse | CTCACGAACTTCACCAGTTTCT |
| ELK3 | Forward | ACCCAAAGGCTTGGAAATCT |
|  | Reverse | TGTATGCTGGAGAGCAGTGG |
| EPS8 | Forward | GATGGAGGAAGTGCAAGATG |
|  | Reverse | GACTGTAACCACGTCTTCACA |
| ETS1 | Forward | TGGAGTCAACCCAGCCTATC |
|  | Reverse | TCTGCAAGGTGTCTGTCTGG |
| FGF2 | Forward | AGTGTGTGCTAACCGTTACCT |
|  | Reverse | ACTGCCCAGTTCGTTTCAGTG |
| FERMT2 | Forward | AAATGGTCACCGTAGAGTTTGC |
|  | Reverse | CTCTCGTTTTGGTCTTTTGCAC |
| HK2 | Forward | CAAAGTGACAGTGGGTGTGG |
|  | Reverse | GCCAGGTCCTTCACTGTCTC |
| IL-1A | Forward | AGATGCCTGAGATACCCAAAACC |
|  | Reverse | CCAAGCACACCCAGTAGTCT |
| IL-1B | Forward | GCACGATGCACCTGTACGAT |
|  | Reverse | CACCAAGCTTTTTTGCTGTGAG |
| IL-6 | Forward | GGTACATCCTCGACGGCATCT |
|  | Reverse | GTGCCTCTTTGCTGCTTTCAC |
| IL-8 | Forward | AGCCTTCCTGATTTCTGCAGCTCT |
|  | Reverse | AATTTCTGTGTTGGCGCAGTGTG |
| IL-33 | Forward | CAAAGAAGTTTGCCCCATGT |
|  | Reverse | AAGGCAAAGCACTCCACAGT |
| LAMC2 | Forward | GATGGCATTCACTGCGAGAAG |
|  | Reverse | TCGAGCACTAAGAGAACCTTTGG |
| NT5E | Forward | TCTTCTAAACAGCAGCATTCC |
|  | Reverse | CATTTCATCCGT GTGTCTCAG |
| GAPDH | Forward | TCCATGACAACTTTGGTATCGTG |
|  | Reverse | ACAGTCTTCTGGGTGGCAGTG |
