# Supplementary material for: PDK1 promotes ovarian cancer metastasis by modulating tumor-mesothelial adhesion, invasion, and angiogenesis via α5β1 integrin and JNK/IL-8 signaling
Source: Oncogenesis. 2020 Feb 18;9(2):24. doi: 10.1038/s41389-020-0209-0 (PMC7028730; doi:10.1038/s41389-020-0209-0)

Supplementary Fig. 1 (A) Transient knockdown of PDK1 (via siPDK1) mRNA and/or protein expression in SKOV-3 and A2780CP, assessed using qPCR (upper panels) and immunoblot (lower panel), respectively. (B) Stable knockdown of PDK1 mRNA and/or protein expression in A2780CP (shPDK1-316 and shPDK1-207) and ES-2 (shPDK1-332 and shPDK1-226), assessed using qPCR (upper panel) and western blot (lower panel), respectively. (C) p-PDHE1α and PDHE1α protein, and fold change of lactate levels in PDK1-silenced (shPDK1-207) and control A2780CP cells determined via immunoblot analysis and lactate colorimetric assay, respectively; Bars: mean ± SD of 3 experiments; *P<0.05; Mann–Whitney test. Media from cultured cells were collected and used for measuring lactate levels.

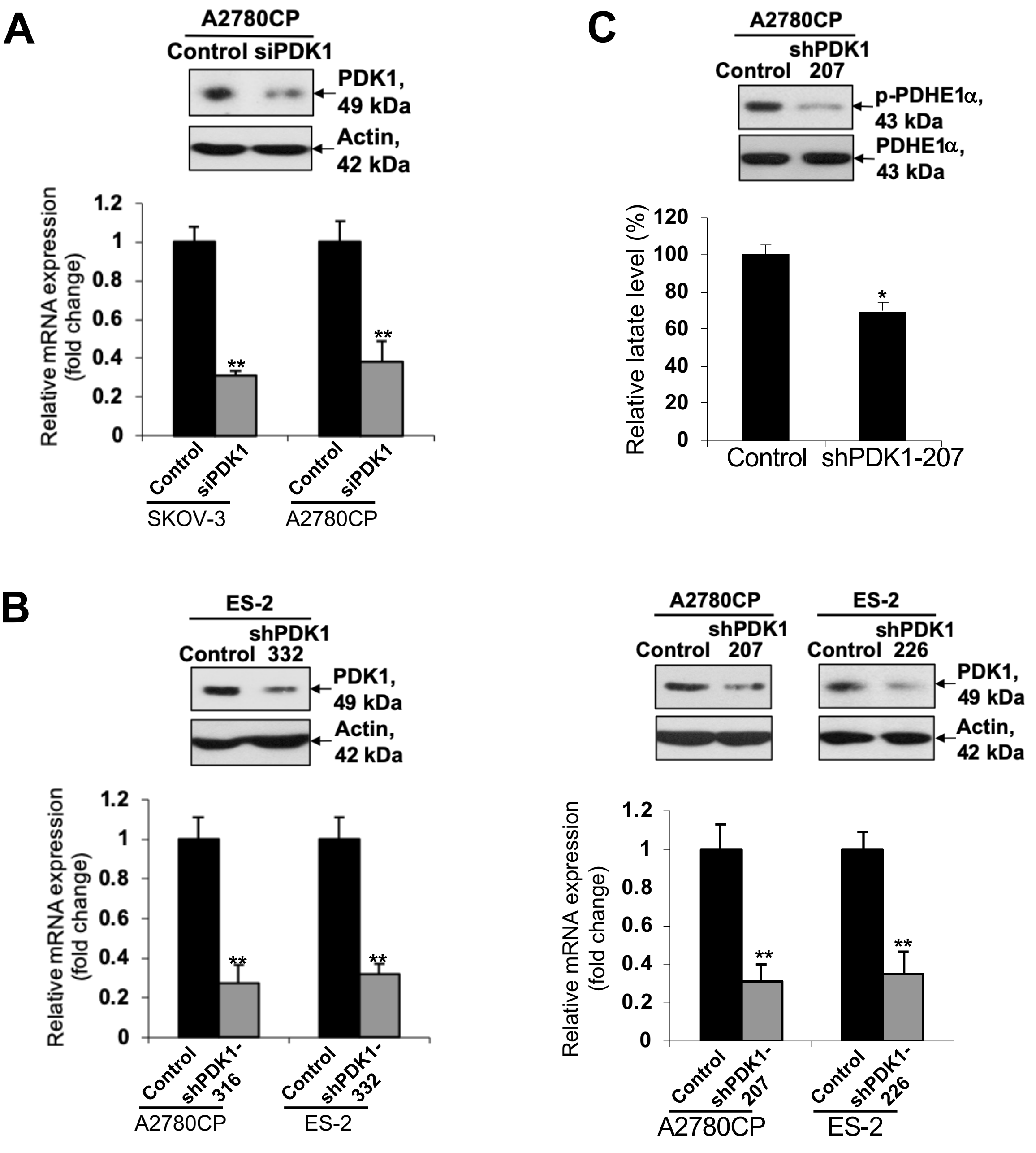

Supplementary Fig. 2 (A) qPCR analysis of α5 and β1 integrin expression in control, shPDK1-207 A2780CP and shPDK1-226 ES-2; Bars: mean ± SD of 3 experiments; *P<0.05; Mann–Whitney test. (B) Specific knockdown of α5 integrin or β1 integrin in OVCAR-3 cells expressing PDK1 transfected with siRNAs of α5 integrin or β1 integrin as detected by immunoblot analyses.

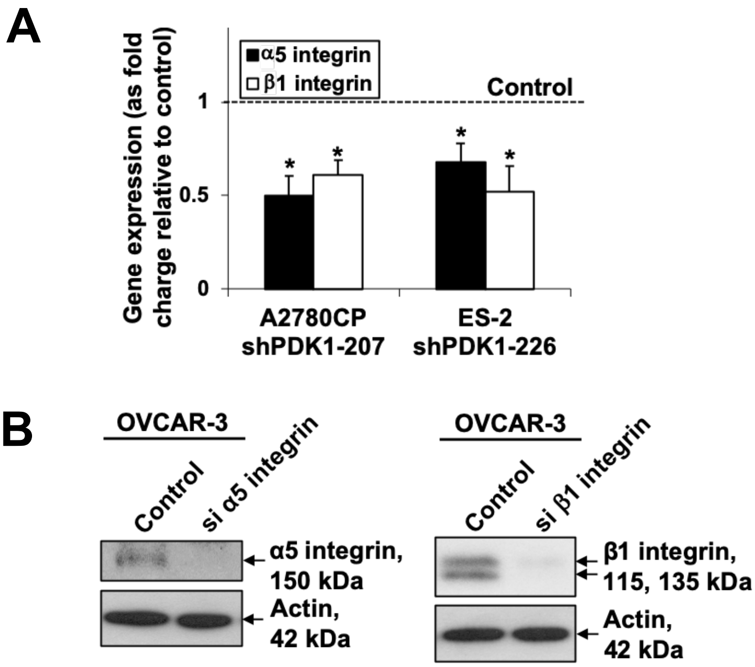

Supplementary Fig. 3 (A) Migration or invasion of SKOV-3 and A2780CP cells with transient knockdown of PDK1 (siPDK1) presented as a percentage of controls; Bars: mean ± SD of 3 experiments; **, P<0.005, Mann–Whitney test. (B) Migration or invasion of A2780CP (shPDK1-207) and ES-2 cells (shPDK1-226) with stable knockdown of PDK1 presented as a percentage of controls; Bars: mean ± SD of 3 experiments; **, P<0.005, Mann–Whitney test. (C) p-PDHE1α and PDHE1α protein expression in DCA-treated and control A2780CP cells determined via immunoblot analysis (left). Migration or invasion of DCA-treated and control A2780CP cells presented as a percentage of controls (right); Bars: mean ± SD of 3 experiments; **, P<0.005, Mann–Whitney test (right).

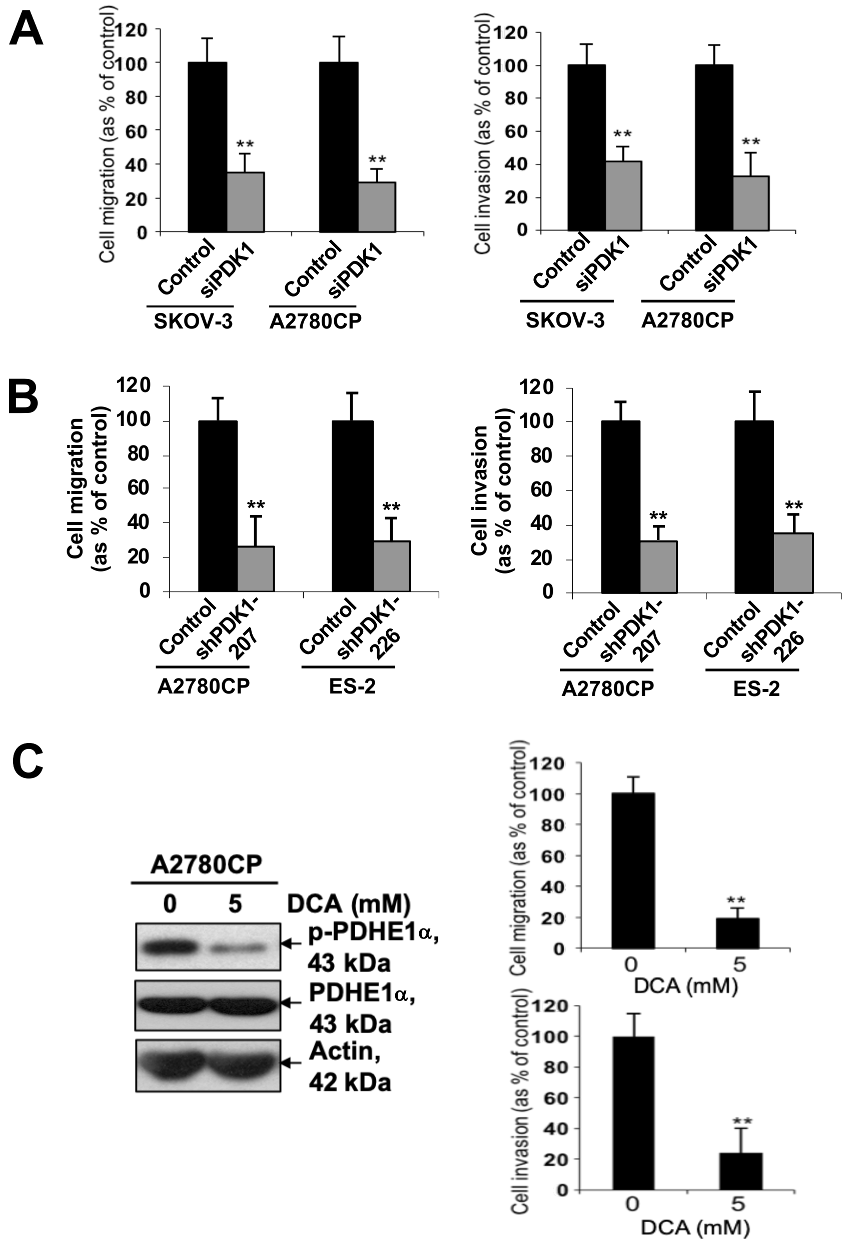

Supplementary Fig. 4 (A) Cell proliferation rate of SKOV-3 and A2780CP in control and siPDK1 (upper) and A2780CP and ES-2 in control and shPDK1(lower) by XTT assay. Cells (2000 cells/well) were seeded in 96-well plates. On day 3, 4 and 5, cell viability was determined using the Cell Proliferation Kit II (Roche, Indianapolis, IN). Bars: mean ± SD of 3 experiments. (B) Cell proliferation rate of A2780CP (left) and ES-2 (right) in control and shPDK1 by cell count method. Cells (3×10^4) were seeded in 12-well or 6-well plates or T150 culture flasks and maintained in growth medium. The cell number was counted using a Luna™ automated cell counter (Logos Biosystems, Annandale, VA) on days 1 (12-well culture plates), 4 (6-well culture plates) and 7 (T150 culture flasks) (C) Soft agar assay in control and stable knockdown of PDK1 in A2780CP. Cells (2×10^4) were suspended in 2 ml medium containing 0.4% agar. The mixture was seeded on a layer of 1% agar in 6-well culture plates. Cells were counted after 18 days. Bars: mean ± SD of 3 experiments; **, P<0.005; Mann–Whitney test.

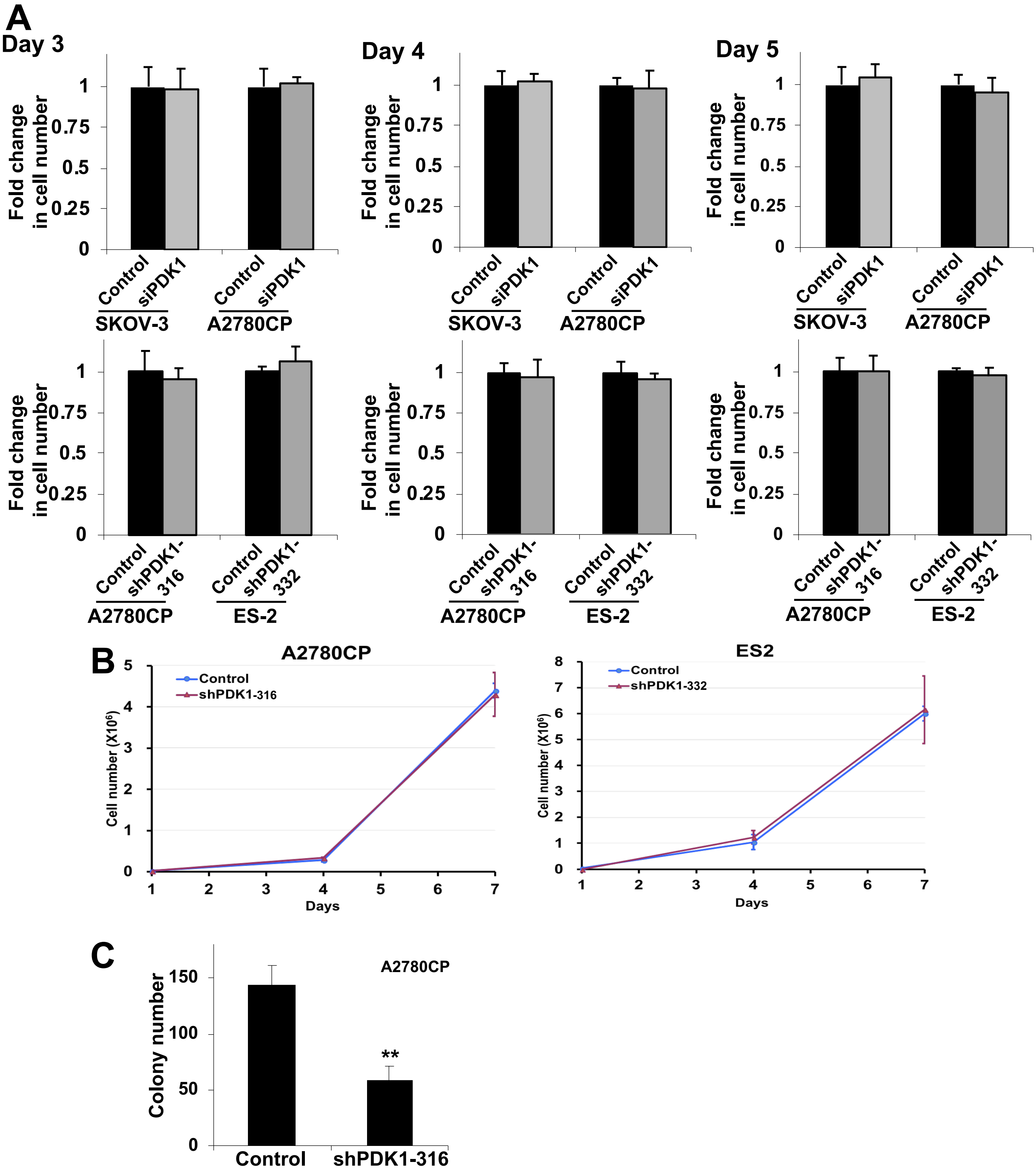

Supplementary Fig. 5 Pathway enrichment analysis of genes (A) downregulated and (B) upregulated after siPDK1 in SKOV-3 cells. (C) Hierarchical clustering heatmap of 16 metastasis-related genes and PDK1 between control and siPDK1 in SKOV-3 cells (Log2FoldChange ≥1 cutoff). (D) mRNA expression of 16 metastasis-related genes, calculated as fold change relative to control in PDK1-silenced A2780CP (shPDK1-207) cells using qPCR; Bars: mean ± SD of 3 experiments; *, P<0.05; **, P<0.005; nd=non-detectable; Mann–Whitney test.

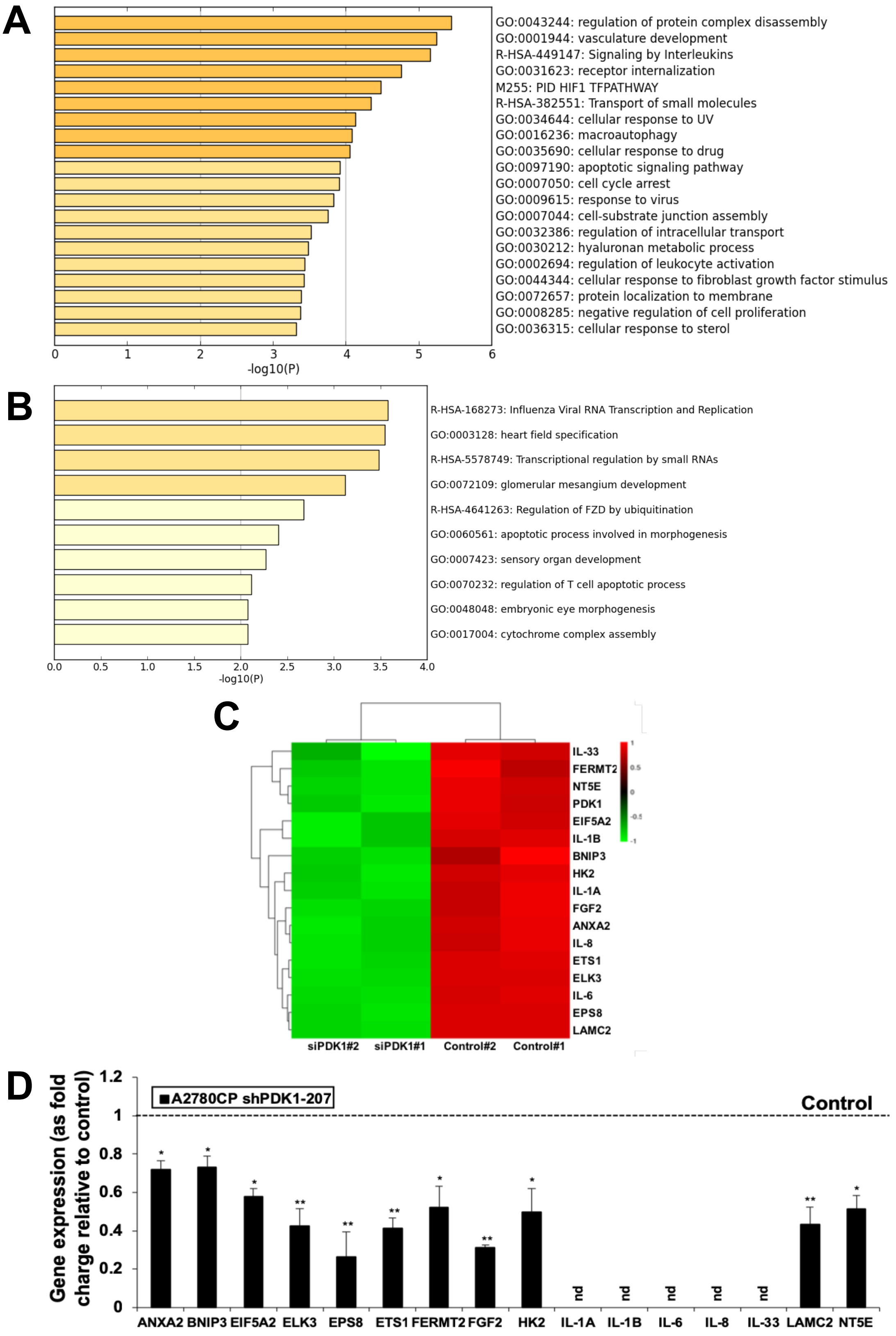

Supplementary Fig. 6 Correlation between metastasis-related genes (BNIP3, EIF5A2, ELK3, EPS8, FERMT2, HK2, IL-1A and IL-6) and PDK1 expression in ovarian cancer clinical samples in TGCA database cohorts by the GEPIA tool.

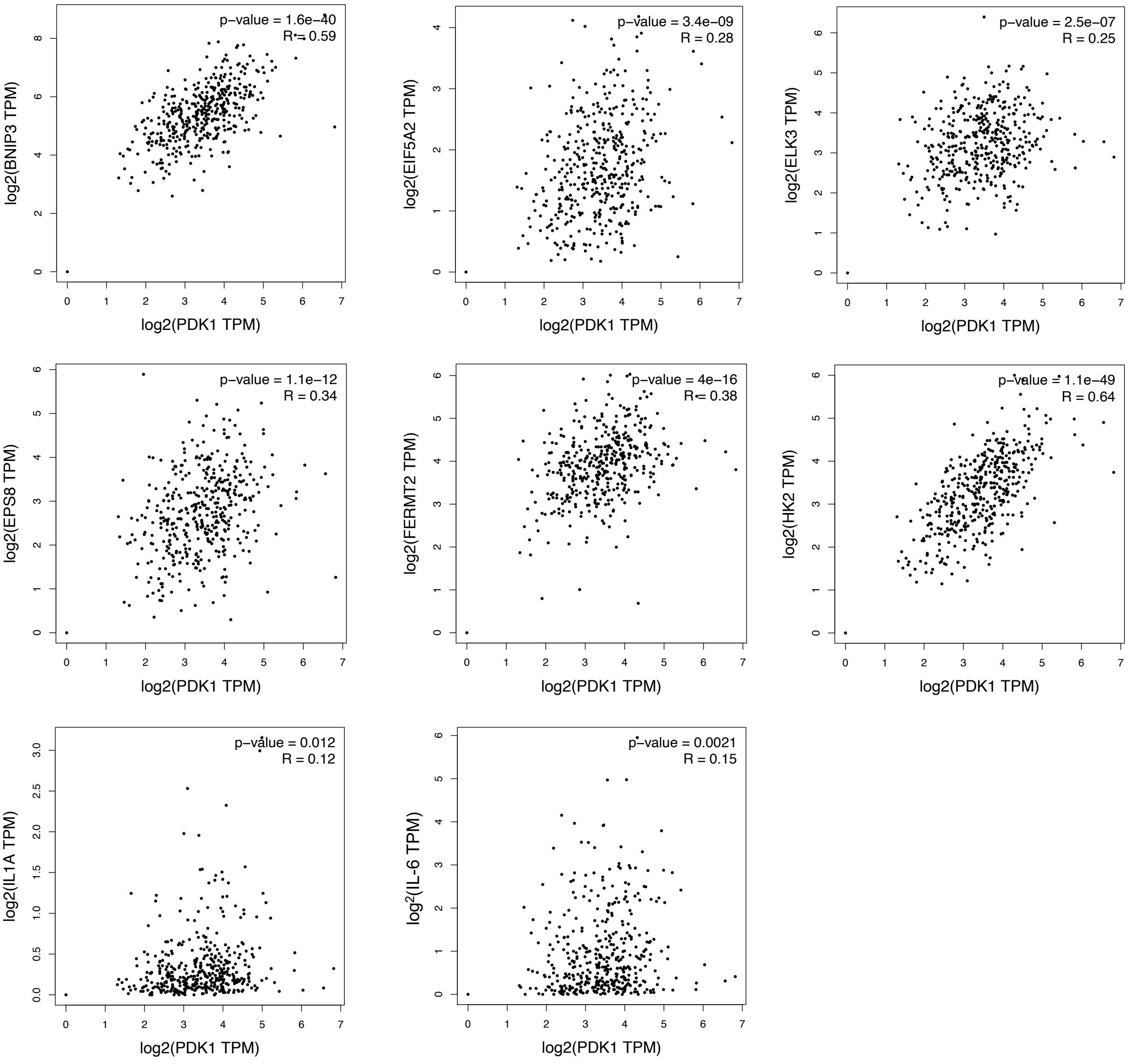

Supplementary Fig. 7 (A) IL-8 mRNA expression and secretion in ES-2 cells with stable knockdown of PDK1 (shPDK-226) via qPCR (left) and ELISA (right), presented as fold change relative to control; Bars: mean ± SD of 3 experiments; *, P<0.05, Mann–Whitney test. (B) Capillary tube formation by human umbilical vein endothelial cells (HUVECs) treated with conditioned medium from ES-2 with stable knockdown of PDK1 (shPDK-226) presented as a percentage of controls; Bars: mean ± SD of 3 experiments; **, P<0.005, Mann–Whitney test. (C) Cell migration and invasion from IL-8-treated ES-2 cells presented as a percentage of controls; Bars: mean ± SD of 3 experiments; *, P<0.05; Mann–Whitney test.

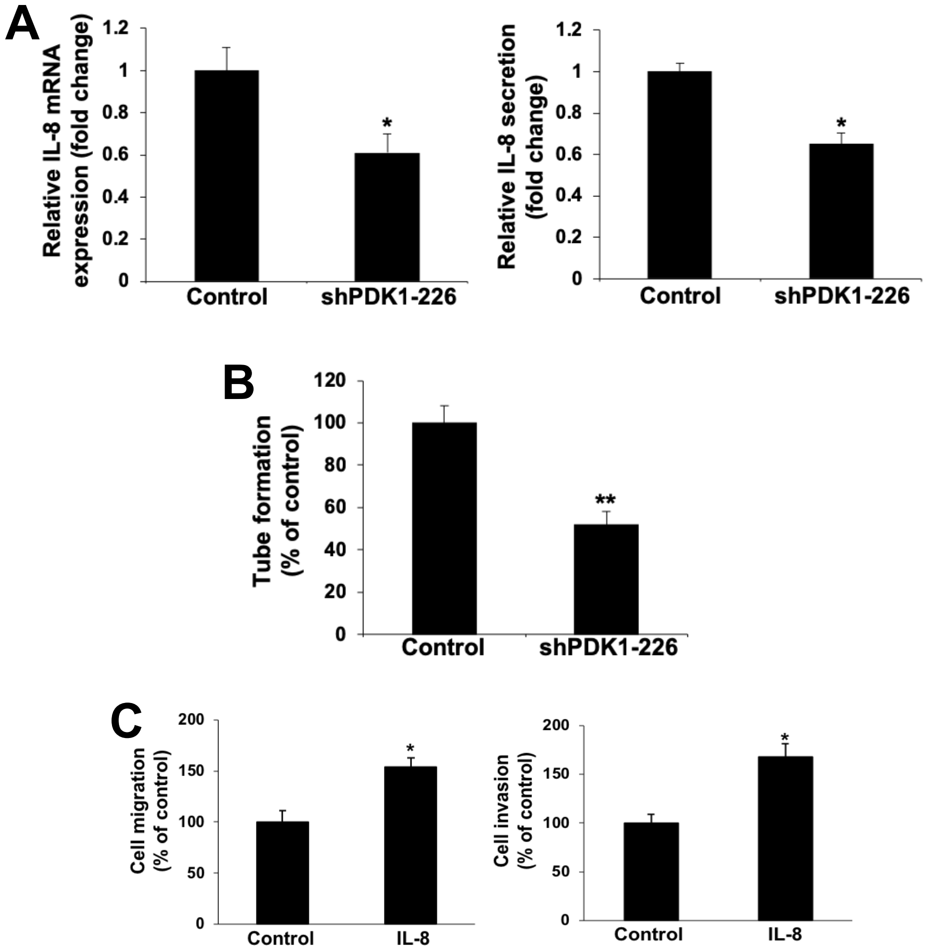

Supplementary Fig. 8 (A) BALB/c female nude mice were injected i.p. (seven mice/group) with ES-2 cells stably depleted of PDK1 (2×10^6). Mice were sacrificed 14 d after cell injection. Tumors were excised and weighed at autopsy. The total i.p. tumor weight in mice inoculated with shPDK1 ES-2 or control cells; Bars: mean ± SD; **, P<0.005, Mann–Whitney test. (B) Excised tumors were snap-frozen for RNA extraction. IL-8 mRNA expression in i.p. tumors from mice inoculated with shPDK1 ES-2 or control cells was determined via qPCR presented as fold change relative to controls; Bars: mean ± SD; *, P<0.05, Mann–Whitney test.

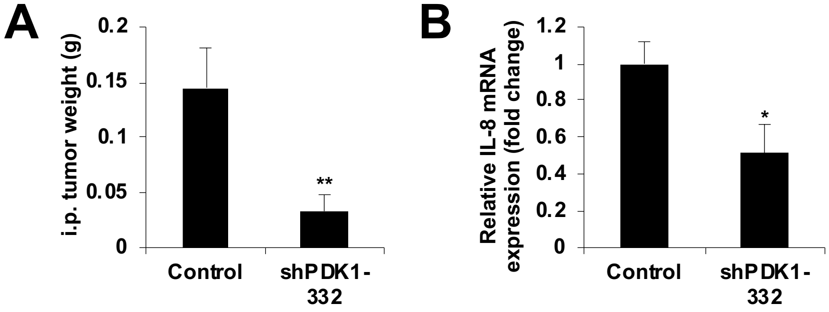

Supplement: Supplementary file 2 — Supp Figs 1-8 [file 41389_2020_209_MOESM2_ESM.pdf]
